# Associations of Skin Biomechanical Properties With Biological Aging Clocks and Longitudinal Changes in Intrinsic Capacity in Adults Aged 20–93: The INSPIRE‐T Project

**DOI:** 10.1111/acel.70190

**Published:** 2025-08-10

**Authors:** Wan‐Hsuan Lu, Sophie Guyonnet, Jérémy Raffin, Sandrine Bessou‐Touya, Katia Ravard Helffer, Pascale Bianchi, Jimmy Le Digabel, Paul Bensadoun, Jean‐Marc Lemaitre, Philipe de Souto Barreto, Bruno Vellas, the IHU HealthAge INSPIRE/Open Science study group, Sophie Guyonnet, Bruno Vellas, Lauréane Brigitte, Agathe Milhet, Elodie Paez, Emeline Muller, Sabine Le Floch, Catherine Takeda, Catherine Faisant, Françoise Lala, Gabor Abellan Van Kan, Zara Steinmeyer, Antoine Piau, Tony Macaron, Davide Angioni, Pierre‐Jean Ousset, Mélanie Comté, Nathalie Daniaud, Fanny Boissou‐Parachaud, Sandrine Andrieu, Christelle Cantet, Yves Rolland, Philipe de Souto Barreto, Fabien Pillard, Bernard Teysseyre, Marie Faruch, Pierre Payoux, Catherine Takeda, Neda Tavassoli, Marie Dorard, Bénédicte Razat, Camille Champigny, Sophie Guyonnet, Cédric Dray, Jean‐Philippe Pradère, Angelo Parini, Yohan Santin, Dominique Langin, Pierre Gourdy, Laurent O. Martinez, Anne Bouloumié, Angelo Parini, Nicolas Fazilleau, Roland Liblau, Jean‐Charles Guéry, Michel Simon, Nicolas Gaudenzio, Luciana Bostan, Hicham El Costa, Nabila Jabrane Ferrat, Philippe Valet, Cedric Dray, Isabelle Ader, Valérie Planat, Louis Casteilla, Pierre Payoux, Patrice Peran, Cyrille Delpierre, Sandrine Andrieu, Claire Rampon, Noelie Davezac, Bruno Guiard, Nathalie Vergnolle, Jean‐Paul Motta, Sara Djebali, Pauline Floch, Céline Deraison, Chrystelle Bonnart, Jean‐Emmanuel Sarry, Nicola Coley, Sophie Guyonnet, Christelle Cantet, Jessica Pontary, Sandrine Andrieu

**Affiliations:** ^1^ IHU HealthAge Toulouse France; ^2^ Institute on Aging Toulouse University Hospital (CHU Toulouse) Toulouse France; ^3^ CERPOP, UMR 1295 Université Paul Sabatier Toulouse France; ^4^ R&D Pierre Fabre Dermo‐Cosmétique & Personal Care Toulouse France; ^5^ INSERM IRMB UMR1183, Hôpital Saint Eloi Université de Montpellier Montpellier France

**Keywords:** biomarkers, biomechanical phenomena, epigenesis, healthy aging, inflammation, intrinsic capacity, skin aging

## Abstract

Evidence connecting skin aging to functional decline and systemic aging biomarkers is lacking. This study investigated how skin‐aging biomechanics were associated with changes in intrinsic capacity (IC), a marker of healthy aging. We also explored their links with biological aging clocks (epigenetic and inflammatory clocks) and potential moderating effects on the skin‐IC relationship. Baseline skin elasticity and viscoelasticity were measured in 441 INSPIRE‐T participants aged 20 to 93 (59.9% women) using Cutometer parameters. IC was evaluated over 3 years as a five‐domain score covering cognition, locomotion, psychology, vitality, and sensory (a higher score indicated better). Biological aging was measured at baseline using six epigenetic clocks (Horvath pan‐tissue, Horvath skin & blood, Hannum, PhenoAge, GrimAge, and DunedinPACE) and inflammatory clock (iAge). Poor skin elasticity and viscoelasticity in older adults were associated with lower baseline IC after controlling for demographic, medical, and lifestyle factors. Longitudinally, older men with a higher viscoelastic ratio (R6) experienced a faster decline in IC (a standardized coefficient [95% CI] ranged from −0.37 [−0.72, −0.03] at age 62 to −1.32 [−1.91, −0.73] at age 93). Accelerated iAge was associated with reduced skin elasticity (R2, R5, R7). Moreover, the association between parameters related to elastic recovery (R5, R7) and baseline IC became more pronounced as accelerated iAge increased. This is the first study demonstrating the association between skin‐aging biomechanics and IC. Poor skin elasticity was associated with higher systemic inflammation. Therefore, skin biomechanical properties may reflect overall functional aging, with inflammation serving as a common underlying factor.

## Graphical abstract

This study assessed skin biomechanics using the Cutometer in 441 community‐dwelling adults aged 20–93 from the INSPIRE‐T cohort. We demonstrated that skin elasticity and viscoelasticity parameters were associated with the intrinsic capacity (IC) score, a functional marker of healthy aging encompassing cognition, locomotion, psychology, vitality, and sensory functions. The relationships between skin parameters and biological aging clocks (epigenetic and inflammatory) were also investigated.

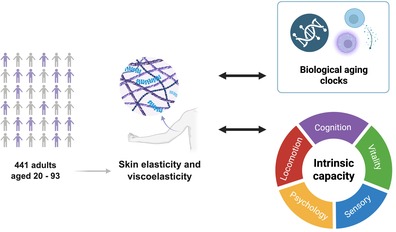

## 1 Introduction

Aging is associated with the gradual accumulation of molecular and cellular damage from early life, manifesting as physiological changes; in advanced stages, biological damage may lead to functional impairments affecting daily life (Ferrucci et al. 2018). To delay or prevent loss of function during aging, the World Health Organization (WHO) elaborated the integrated care for older people (ICOPE; World Health Organization 2019), a function‐centered healthcare pathway dedicated to maximizing intrinsic capacity (IC) – the ensemble of all physical and mental capacities of an individual (Beard et al. 2016) – throughout older adulthood. IC comprises key body domains contributing to healthy aging: cognition, locomotion, psychology, vitality, and sensory (Cesari et al. 2018). The decline in IC can be detected earlier than disability in older adults (Sánchez‐Sánchez et al. 2024), allowing for timely interventions to reverse functional decline in the early stages (Beard et al. 2016; de Souto Barreto et al. 2024). IC is also important in basic research to understand the cellular and molecular mechanisms underlying functional decline during aging (de Souto Barreto et al. 2023).

Aging of the skin is the most visible sign of human aging, commonly characterized by wrinkling, sagging, dryness, and loss of elasticity (Wong and Chew 2021; Gruber et al. 2020). Intrinsic skin aging refers to the inevitable physiological alterations over time, in contrast to damages caused by environmental exposure (i.e., extrinsic skin aging; Csekes and Račková 2021). At the cellular level, intrinsic skin aging involves reduced cell proliferation caused by stem cell dysfunction and attrition, along with the accumulation of senescent dermal fibroblasts and epidermal keratinocytes (Russell‐Goldman and Murphy 2020; Gruber et al. 2020). The structural integrity of the extracellular matrix also deteriorates, mainly resulting from age‐related reduction in collagen synthesis as well as the increase in enzymatic degradation and fragmentation of both collagen and elastin fibers (Wilkinson and Hardman 2021; Lee et al. 2021). Notably, biological mechanisms underlying the changes in intrinsically aged skin, particularly cellular senescence, epigenetic alteration, and inflammation (Gruber et al. 2020; D'Arino et al. 2023; Lemaitre 2024), are also implicated in age‐associated deteriorations and diseases across multiple tissues and organs (Kennedy et al. 2014; López‐Otín et al. 2023). The skin thus represents an ideal model for studying systematic biological aging due to its ease of sampling and phenotyping (Holzscheck et al. 2020). For example, skin elasticity and viscoelasticity, attributed to the stability of the elastin and collagen fiber network, significantly change with aging and can be assessed using noninvasive methods. However, evidence supporting a direct relationship between skin aging and age‐related phenotypes/disorders remains limited (Zouboulis and Makrantonaki 2019). To the best of our knowledge, no study has explored functional outcomes, including IC—an indicator of healthy aging.

In the present study, we aimed to investigate the association between skin biomechanical properties, serving as a marker of skin aging, and functional aging across adulthood. This study had two main objectives. First, we investigated if skin elasticity and viscoelasticity, measured using Cutometer parameters, were associated with IC over three years in community‐dwelling adults aged from 20 to 93 years. Second, we evaluated the association of skin elasticity and viscoelasticity with biological aging, measured through epigenetic clocks (Oblak et al. 2021) and the inflammatory clock (Sayed et al. 2021). As an exploratory analysis, we examined whether biological aging moderated the skin‐IC relationship.

## 2 Methods

### 2.1 Study Population

The current study used data from the Human Translational Research Cohort of the INSPIRE program (ClinicalTrials.gov [NCT04224038]; version 1.0 of the INSPIRE‐T database), which is an ongoing, observational, and longitudinal cohort established in 2019 to explore biomarkers of aging, age‐related conditions, and IC evolution in humans (de Souto Barreto et al. 2021; Guyonnet et al. 2020). The details of the INSPIRE‐T cohort are provided in Supporting Information—Methods. Until 2024, the INSPIRE‐T program has included 1132 subjects, most completing up to 3 years of follow‐up visits. Among them, 518 people recruited before April 2021 received Cutometer measurements at baseline on a voluntary basis. Data from 77 individuals were excluded due to poor quality, as evaluated by a skin data specialist unaffiliated with the nurses responsible for Cutometer assessments. Finally, a sample of 441 individuals (aged 20–93) was used for analysis. Within this population, 440 subjects had available data on IC over 3 years. Data on blood‐derived epigenetic clocks and inflammatory clock were accessible for 434 and 436 subjects, respectively (Figure S1).

### 2.2 Skin Biomechanical Properties

Skin elasticity and viscoelasticity were measured on the day of study enrollment using Cutometer Dual MPA 580 (Courage & Khazaka Electronic GmbH, Cologne, Germany) with a 2‐mm‐diameter probe. Participants sat with the probe placed on the inner side of their upper arm (sun‐unexposed area). A 450‐mbar vacuum was applied to evaluate the skin deformation; the assessment comprised five successive cycles, each including a 5‐s suction followed by 5 s of relaxation. The software of Cutometer later generated mechanical parameters based on data from five cycles.

We analyzed five Cutometer parameters frequently investigated in prior studies (Ahn et al. 2007; Fujimoto et al. 2023; Huang et al. 2024): (1) Skin distensibility—R0, the total distension during the suction phase, measured in mm. (2) Gross elasticity—R2, the ratio of final recovery (at the end of the relaxation) to total distension. (3) Net elasticity—R5, which excludes viscous distension and recovery compared to R2. (4) Biological elasticity—R7, the ratio of immediate recovery (within the first 0.1 s during the relaxation phase) to total distension, excluding viscous recovery compared to R2. (5) Viscoelastic ratio—R6, the ratio of viscoelastic to elastic distension.

R2, R5, and R7 are markers of skin elasticity, with higher values indicating better elasticity; R5 and R7 primarily reflect the extent of elastic recovery, whereas R2 encompasses both elastic and viscous recovery. R6 serves as the parameter for viscoelasticity, representing the proportion of viscous deformation during the suction phase, which usually increases with age. Unlike R0, the parameters R2, R5, R6, and R7 are dimensionless and independent of skin thickness, enabling a direct comparison between individuals (Abbas et al. 2022). Moreover, R2, R5, R6, and R7 were suggested as indicators of “skin elasticity age” due to their strong correlation with age compared to other R parameters from Cutometer (Ryu et al. 2008).

### 2.3 Intrinsic Capacity (IC)

IC was operationalized as a composite score of five functional domains involving cognition (assessed by the Mini‐Mental State Examination [MMSE] (Folstein et al. 1975)), locomotion (measured by the Short Physical Performance Battery [SPPB] (Guralnik et al. 1994)), psychology (examined by the 9‐item Patient Health Questionnaire for depression [PHQ‐9] (Kroenke et al. 2001)), vitality (evaluated as grip strength in the dominant hand), and sensory (including distance vision assessment and whisper test). Each domain was assessed by trained staff at baseline and then annually for up to 3 years. Following the methods in the prior INSPIRE‐T publication (Lu et al. 2023), the original scores from the domain assessments were rescaled to a range of 0–100 points, with higher indicating better performance. The final IC score was calculated as the average of the five domains.

### 2.4 Biological Aging Clocks

Six epigenetic clocks were examined at baseline, including first‐generation clocks (Horvath pan‐tissue, Horvath skin & blood, Hannum; trained on chronological age), second‐generation clocks (PhenoAge and GrimAge; trained on lifespan and mortality risk), and a third‐generation clock (DunedinPACE; trained on pace of aging; Oblak et al. 2021). Details of epigenetic clock assessments are provided in Supporting Information—Methods. For the first‐ and second‐generation clocks, we calculated age acceleration (AA) for each individual, defined as the residuals from regressing chronological age against DNA methylation ages (DNAmAge). A positive AA value indicated that epigenetic age exceeded chronological age, whereas a negative AA value indicated that epigenetic age was lower than chronological age. DunedinPACE reflected the pace of biological aging per year of time; a value > 1 indicated accelerated biological aging.

The inflammatory clock (iAge), developed by Sayed et al. (2021), measures age‐related systemic inflammation using a set of blood immune molecules identified through a deep learning method. In this study, iAge was estimated from the baseline blood sample following the procedures outlined in Sayed et al.'s work. To align with epigenetic AA, we calculated residuals from the regression of chronological age and iAge (referred hereafter as iAgeAA), where positive values indicated an estimated iAge exceeding chronological age.

### 2.5 Covariates

Factors influencing individuals' skin elasticity were collected at the baseline visit, including age, sex (Firooz et al. 2012), hormone replacement therapy (HRT) use (Sator et al. 2001), and long‐term sun exposure (Bernstein et al. 1996). HRT users were identified if participants reported taking oral or topical HRT medications due to menopause symptoms. Factors related to sun exposure included previous residence in high daily solar exposure regions, use of ultraviolet (UV) protection products, and the frequency of sun exposure on the lower limbs (detailed in Supporting Information—Methods).

### 2.6 Statistical Analysis

#### 2.6.1 Descriptive Analysis

We performed descriptive statistics on the participants' characteristics, R parameters, and biological aging clocks; the results were presented as means with standard deviations (SDs) or as numbers with percentages, as appropriate. Correlations between age and R parameters were illustrated using scatter plots and analyzed using Spearman's rank order correlation coefficients.

#### 2.6.2 Association Between R Parameters and IC

We applied linear mixed‐effect models with random intercepts and random time slopes at the participants' level. Each R parameter was analyzed in a separate model as a predictor. To allow for comparison across models, all R parameters were standardized (mean = 0, SD = 1), such that coefficients could be interpreted as the effect of a one‐SD increase in the R parameter on IC.

We ran both unadjusted models (including only R, time, and their interaction as independent variables) and adjusted models that controlled for age, sex, and the aforementioned covariates. Since both R parameters and IC vary with age, it is plausible that their associations also change throughout the lifespan. Therefore, interaction terms involving time, the R parameter, and age were tested in the adjusted models; significant interaction terms were retained, and their coefficients and *p* values were reported.

We took two approaches to facilitate the interpretation of models with significant age interactions. First, we applied the Johnson‐Neyman technique (Johnson and Fay 1950; Hayes and Matthes 2009) to identify age intervals where the association between R and IC was statistically significant, and then reported the conditional effects of R on IC at the lower and upper boundaries of this interval. Second, we created interaction plots to visualize the effect of R on IC, holding age constant at 40, 65, and 80 years, respectively. These age points were selected to represent young, old, and very old adults.

All analyses regarding IC were conducted on the whole population and then separately by sex to explore potential sex differences.

#### 2.6.3 Association Between R Parameters and Biological Aging Clocks

The association of R parameters with epigenetic aging and iAgeAA was evaluated using linear regression. A total of 35 models were examined, each testing a separate R parameter (as a dependent variable) and biomarker (as a predictor). Results from both unadjusted models and models adjusted for age, age^2^ (when appropriate), sex, and covariates were reported. Furthermore, to test whether the associations between biological aging clocks and R parameters varied by age or sex, we included interaction terms between biomarkers and age as well as between biomarkers and sex in the linear models. To ensure comparability across models, standardized regression coefficients were reported, indicating the expected change (in SD units) in the R parameter for each one SD increase in epigenetic aging or iAgeAA.

#### 2.6.4 Exploratory Analyses

We conducted two exploratory analyses. First, we ran linear mixed‐effect models using each IC domain score (on a scale of 0–100 points) as an outcome to investigate the effects of R parameters on individual IC domains. Second, given that some R parameters were significantly associated with both IC and biological aging clocks, we investigated whether the association between R parameters and IC was moderated by biological aging. To do so, we added biological aging clocks and their interaction terms to the mixed‐effect models previously applied to assess the R–IC association. Interaction plots were used to illustrate the R–IC relationship at different levels of biological aging, as defined by quartiles; the plots held age constant at 40, 65, or 80 years, respectively, to highlight potential age differences. This exploratory analysis was conducted only for the R parameters and biomarkers that showed statistical significance in the main analyses.

Our analyses were performed using SAS version 9.4 (SAS Institute Inc., Cary, NC) and R Studio version 2024.9.0.375, with a significance level of 0.05. Listwise deletion was applied to observations with any missing data on the variables required for regression models (IC, biomarkers, or covariates).

## 3 Results

### 3.1 Descriptive Analysis of Population Characteristics and Skin Biomechanics

**FIGURE 1:**
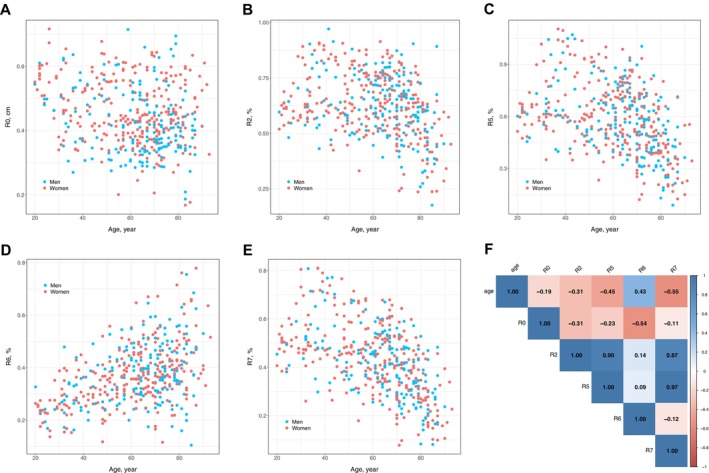
Correlations between skin elasticity/viscoelasticity parameters and age. (A–E) Scatterplots show how Cutometer R parameters varied by age. Blue points represent men, and red points represent women. R0, skin distensibility; R2, gross elasticity; R5, net elasticity; R6, viscoelastic ratio; R7, biological elasticity. (F) A correlation matrix representing Spearman's rank correlation coefficients between age and R parameters. All *p* values for the correlations were statistically significant (*p* < 0.05), except for the comparison between parameters R5 and R6 (*p* = 0.05).

Table 1 and Table S1 display the baseline characteristics of 441 participants, with a mean (SD) age of 61.3 (17.5) years and the majority being women (59.9%). Figure 1 presents the distribution of R parameters across age (Figure 1A–E) and their Spearman's rank correlation coefficients (Figure 1F). R0 (skin distensibility) showed a weak correlation with age (*r* = −0.19; *p* < 0.001) and did not follow a consistent trend (Figure 1A). Skin elasticity parameters, including R2 (gross elasticity), R5 (net elasticity), and R7 (biological elasticity), exhibited significant negative correlations with age (Figure 1B,C,E), with Spearman's rank correlation coefficients ranging from −0.31 to −0.56 (all *p* < 0.001; Figure 1F). In contrast, R6 (viscoelastic ratio) tended to increase in older age (*r* = 0.43; *p* < 0.001; Figure 1D).

**TABLE 1:** Baseline characteristics of 441 INSPIRE‐T participants with skin elasticity and viscoelasticity data.

	Whole population (*n* = 441)	Women (*n* = 264 [59.9%])	Men (*n* = 177 [40.1%])
Age, year	61.3 (17.5)	59.9 (18.2)	63.4 (16.3)
Baseline IC score, 0–100 (*n* = 431)	85.5 (7.4)	85.2 (7.7)	86.0 (7.1)
Skin Cutometer parameters			
R0—skin distensibility, mm	0.45 (0.10)	0.46 (0.10)	0.42 (0.10)
R2—gross elasticity, %	0.63 (0.14)	0.63 (0.14)	0.62 (0.14)
R5—net elasticity, %	0.58 (0.20)	0.59 (0.20)	0.57 (0.19)
R6—viscoelastic ratio, %	0.37 (0.11)	0.37 (0.11)	0.37 (0.12)
R7—biological elasticity, %	0.42 (0.15)	0.43 (0.15)	0.41 (0.14)
Biological aging clocks			
HorvathAA pan‐tissue (*n* = 434)	−0.45 (4.47)	−1.23 (4.42)	0.71 (4.31)
HorvathAA skin‐blood (*n* = 434)	0.03 (3.22)	−0.35 (3.26)	0.60 (3.07)
HannumAA (*n* = 434)	−0.11 (3.34)	−0.66 (3.25)	0.71 (3.30)
PhenoAA (*n* = 434)	−0.35 (4.82)	−0.68 (4.62)	0.15 (5.08)
GrimAA (*n* = 434)	0.53 (3.49)	−0.54 (3.09)	2.11 (3.46)
DunedinPACE (*n* = 434)	0.93 (0.11)	0.93 (0.12)	0.93 (0.11)
iAgeAA (*n* = 436)	−0.62 (8.32)	−0.13 (8.28)	−1.34 (8.36)

*Note:* Values are displayed as mean (SD).

Abbreviations: AA, age acceleration obtained using the corresponding biological aging clocks (unit: year); IC, intrinsic capacity.

### 3.2 Associations Between Skin Biomechanics and IC

In unadjusted analyses, we observed significant associations between IC and the R parameters, except for R0 (skin distensibility) (Table 2). Higher values of R2, R5, and R7, which indicate better skin elasticity, were associated with higher IC scores at baseline (R2: β [SE] = 1.83 [0.33], *p* < 0.001; R5: β [SE] = 2.07 [0.32], *p* < 0.001; R7: β [SE] = 2.50 [0.32], *p* < 0.001). Longitudinally, better skin elasticity predicted improvement in IC over time (time × R2: β [SE] = 0.30 [0.10], *p* = 0.004; time × R5: β [SE] = 0.31 [0.10], *p* = 0.003; time × R7: β [SE] = 0.37 [0.10], *p* < 0.001) (Table 2). On the other hand, a higher R6 (viscoelastic ratio) was associated with lower baseline IC (R6: β [SE] = −2.23 [0.32], *p* < 0.001) and a faster IC decline over time (time × R6: β [SE] = −0.30 [0.10], *p* = 0.005) (Table 2).

**TABLE 2:** Linear mixed‐effects model results for IC, based on the whole population.

	R0 (skin distensibility)			R2 (gross elasticity)			R5 (net elasticity)			R6 (viscoelastic ratio)			R7 (biological elasticity)		
	β	SE	*p*	β	SE	*p*	β	SE	*p*	β	SE	*p*	β	SE	*p*
Unadjusted model															
R	0.59	0.34	0.079	1.83	0.33	< 0.001	2.07	0.32	< 0.001	−2.23	0.32	< 0.001	2.50	0.32	< 0.001
Time	−0.15	0.10	0.145	−0.16	0.10	0.120	−0.15	0.10	0.132	−0.15	0.10	0.136	−0.15	0.10	0.132
Time × R	0.05	0.10	0.620	0.30	0.10	0.004	0.31	0.10	0.003	−0.30	0.10	0.005	0.37	0.10	< 0.001
Adjusted model^a^															
R	−0.18	0.29	0.521	−4.79	1.03	< 0.001	−5.43	0.99	< 0.001	3.69	1.14	0.001	−6.21	0.96	< 0.001
Age	−0.23	0.02	< 0.001	−0.20	0.02	< 0.001	−0.21	0.02	< 0.001	−0.24	0.02	< 0.001	−0.22	0.02	< 0.001
R × age	—^b^	—	—	0.08	0.02	< 0.001	0.09	0.02	< 0.001	−0.06	0.02	< 0.001	0.11	0.01	< 0.001
Time	1.91	0.39	< 0.001	1.44	0.38	< 0.001	1.44	0.40	< 0.001	2.27	0.46	< 0.001	1.35	0.42	0.001
Time × R	−0.81	0.35	0.020	0.17	0.11	0.101	0.12	0.11	0.266	1.29	0.42	0.002	0.15	0.12	0.205
Time × age	−0.03	0.01	< 0.001	−0.03	0.01	< 0.001	−0.03	0.01	< 0.001	−0.04	0.01	< 0.001	−0.02	0.01	< 0.001
Time × R × age	0.01	0.01	0.023	—^c^	—	—	—^c^	—	—	−0.02	0.01	0.001	—^c^	—	—

*Note:* Analyses were conducted on 440 individuals for unadjusted models and on 431 individuals for adjusted models. The values of R parameters were first standardized to have a mean of 0 and a SD of 1 before performing the linear mixed‐effect models, allowing for comparisons across models. A year was used as the unit of time.

^a^ The models were additionally adjusted for sex, use of hormone replacement therapy (HRT), previous residence in high‐solar‐exposure regions, use of ultraviolet (UV) protection products, and sun exposure frequency.

^b^ The interaction term “R0 × age” was not retained in the model due to its insignificance.

^c^ The interaction terms “time × R2 × age,” “time × R5 × age,” “time × R7 × age” were not retained in the models due to their insignificance.

**FIGURE 2:**
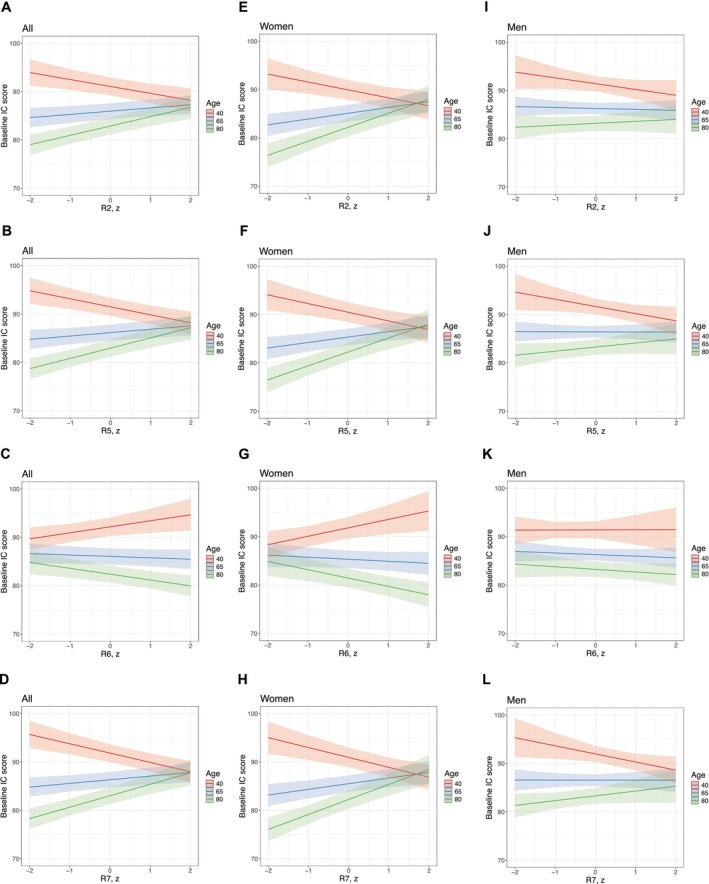
Effect of skin elasticity (R2, R5, R7) and viscoelasticity (R6) parameters on baseline IC at ages 40, 65, and 80. The interaction plots illustrate how the effects of Cutometer R parameters on baseline IC scores varied by age, using 40, 65, and 80 years as examples. Results are presented for the whole population (A–D), women (E–H), and men (I–L). The slopes of R parameters were estimated by holding time = 0 and ages = 40, 65, and 80, respectively, using the linear mixed‐effect models in Table 2 (for the whole population) and Table 3 (for women and men). Overall, the association between baseline IC and R parameters differed between younger (age 40; in red) and older individuals (age 65 and 80; in blue and green), as indicated by the opposite slope directions in the plots. R2, gross elasticity; R5, net elasticity; R6, viscoelastic ratio; R7, biological elasticity.

R2, R5, R6, and R7 remained associated with baseline IC scores after adjusting for covariates, with significant interactions between age and R (Table 2). The Johnson‐Neyman technique revealed that the associations between R and baseline IC were significant only at younger and older ages (Table S2). Figure 2A–D illustrates the age‐moderating association between the R parameters and baseline IC, presenting ages 40, 65, and 80 as examples: the relationship between R and baseline IC at age 40 was in the opposite direction compared to those at ages 65 and 80.

**FIGURE 3:**
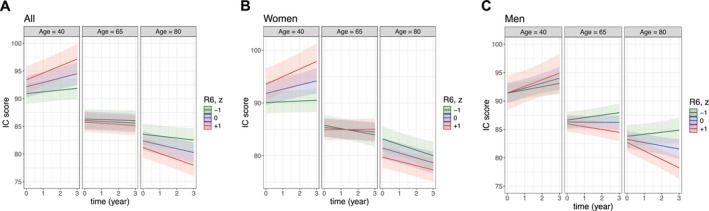
Predicted IC over 3 years by different levels of R6 and ages. The interaction plots illustrate how the effect of the viscoelastic ratio R6 on the overtime IC change varied by ages, using age = 40, 65, and 80 years and standardized R6 values = −1, 0, and 1 as examples. The slopes of time (referring to the overtime IC change) were estimated using the linear mixed‐effect models in Table 2 (for the whole population) and Table 3 (for women and men). (A) IC tended to be improved over time in individuals aged 40 (positive slopes in the plots) and relatively stable or declining at ages 65 and 80. (B, C) Women aged 40 with increased R6 were associated with a higher IC initially and a greater improvement in IC over three years. On the contrary, in older people, especially men, an increased R6 was associated with a lower baseline IC and a faster IC decline.

R6 (viscoelastic ratio) was longitudinally associated with IC change in the adjusted model, and this association was moderated by age (time × R6: β [SE] = 1.29 [0.42], *p* = 0.002; time × R6 *×* age: β [SE] = −0.02 [0.01]; *p* = 0.001; Table 2). Higher R6 was associated with a faster decline in IC at older ages (from 75 to 93 years), with the conditional effect on the IC change ranging from −0.25 (95% CI, −0.50 to −0.01) at age 75 to −0.62 (−1.01 to −0.23) at age 93. In contrast, opposite associations between R6 and overtime IC change were found at age ≤ 47 (Table S3). Figure 3A presents how the impact of R6 on IC trajectories differs at ages 40, 65, and 80 in the whole population. Skin distensibility (R0) was also associated with overtime IC change after adjusting for covariates (time × R0: β [SE] = −0.81 [0.35], *p* = 0.020; time × R0 × age: β [SE] = 0.01 [0.01], *p* = 0.023; Table 2). This association was mainly observed in adults aged ≤ 43, with an increased R0 being associated with worse IC over time (β [95% CI] ranged from −0.56 [−1.05, −0.08] at age 20 to −0.28 [−0.56, −0.00] at age 43; Table S3).

In the sex‐stratified analysis, IC was cross‐sectionally associated with R5 (net elasticity) and R7 (biological elasticity) in both women and men, whereas significant associations with R2 (gross elasticity) and R6 (viscoelastic ratio) were observed only in women (Table 3, Table S2, Figure 2E–2L). A higher R6 predicted a faster IC decline in older men (β [95% CI] ranging from −0.37 [−0.72, −0.03] at age 62 to −1.32 [−1.91, −0.73] at age 93) but not in older women (Table S3, Figure 3B–C). The results of the exploratory analysis on individual IC domains were consistent with the main findings. Skin elasticity parameters R2, R5, and R7 were cross‐sectionally associated with all IC domains. Longitudinally, associations with R6 were primarily observed in the cognition, locomotion, and sensory domains (Table S4).

**TABLE 3:** Linear mixed‐effects model results for IC, stratified by sex.

	R0 (skin distensibility)			R2 (gross elasticity)			R5 (net elasticity)			R6 (viscoelastic ratio)			R7 (biological elasticity)		
	β	SE	*p*	β	SE	*p*	β	SE	*p*	β	SE	*p*	β	SE	*p*
Women (*n* = 259)^a^															
R	−0.08	0.39	0.826	−6.17	1.30	< 0.001	−6.48	1.23	< 0.001	5.20	1.45	< 0.001	−7.19	1.19	< 0.001
Age	−0.24	0.02	< 0.001	−0.19	0.02	< 0.001	−0.21	0.02	< 0.001	−0.26	0.03	< 0.001	−0.22	0.02	< 0.001
R × age	—^b^	—	—	0.11	0.02	< 0.001	0.12	0.02	< 0.001	−0.09	0.02	< 0.001	0.13	0.02	< 0.001
Time	1.90	0.50	< 0.001	1.30	0.45	0.004	1.32	0.47	0.005	2.52	0.58	< 0.001	1.39	0.51	0.006
Time × R	−0.75	0.45	0.096	0.16	0.13	0.229	0.10	0.14	0.472	1.17	0.51	0.023	0.04	0.14	0.778
Time × age	−0.03	0.01	< 0.001	−0.02	0.01	0.001	−0.03	0.01	0.001	−0.04	0.01	< 0.001	−0.03	0.01	0.001
Time × R × age	0.01	0.01	0.210	—^c^	—	—	—^c^	—	—	−0.01	0.01	0.085	—^c^	—	—
Men (*n* = 172)^d^															
R	−0.21	0.42	0.619	−2.80	1.64	0.088	−3.82	1.64	0.020	0.58	1.84	0.752	−4.34	1.64	0.008
Age	−0.21	0.03	< 0.001	−0.20	0.03	< 0.001	−0.21	0.03	< 0.001	−0.20	0.03	< 0.001	−0.22	0.03	< 0.001
R × age	—^b^	—	—	0.04	0.02	0.098	0.06	0.02	0.018	−0.01	0.03	0.590	0.07	0.02	0.007
Time	1.98	0.65	0.002	1.79	0.67	0.007	1.74	0.70	0.013	2.29	0.74	0.002	1.35	0.74	0.069
Time × R	−0.80	0.57	0.161	0.20	0.17	0.250	0.17	0.19	0.366	1.51	0.70	0.031	0.36	0.20	0.080
Time × age	−0.03	0.01	0.002	−0.03	0.01	0.003	−0.03	0.01	0.007	−0.04	0.01	0.001	−0.02	0.01	0.046
Time × R × age	0.02	0.01	0.085	—^c^	—	—	—^c^	—	—	−0.03	0.01	0.003	—^c^	—	—

*Note:* The values of R parameters were first standardized to have a mean of 0 and a SD of 1 before performing the linear mixed‐effect models, allowing for comparisons across models. A year was used as the unit of time.

^a^ The models were additionally adjusted for use of hormone replacement therapy (HRT), previous residence in high‐solar‐exposure regions, use of ultraviolet (UV) protection products, and sun exposure frequency.

^b^ The interaction term “R0 × age” was not retained in the model due to its insignificance.

^c^ The interaction terms “time × R2 × age,” “time × R5 × age,” “time × R7 × age” were not retained in the models due to their insignificance.

^d^ The models were additionally adjusted for previous residence in high‐solar‐exposure regions, use of ultraviolet (UV) protection products, and sun exposure frequency.

### 3.3 Association Between Skin Biomechanics and Biological Aging Clocks

Table S5 presents the association between R parameters and biological aging based on epigenetic clocks and iAge. We observed inconsistent results for R0 (skin distensibility). After adjusting for covariates, AA in first‐generation epigenetic clocks showed a positive association with R0 (HorvathAA pan‐tissue: S.β = 0.12, *p* = 0.014; HorvathAA skin‐blood: S.β = 0.12, *p* = 0.023; HannumAA: S.β = 0.09, *p* = 0.053). In contrast, GrimAA, a second‐generation clock, exhibited a reverse association with R0, although it did not reach statistical significance (S.β = −0.10; *p* = 0.056). A trend between higher DunedinPACE and increased R6 (viscoelastic ratio) was observed without reaching statistical significance (S.β = 0.08, *p* = 0.062), indicating that a faster pace of biological aging was associated with poor viscoelasticity. Finally, individuals with iAgeAA were associated with poor skin elasticity, indicating decreased R2, R5, and R7 (iAgeAA: standardized regression coefficient [S.β] = −0.10, *p* = 0.018 for R2; S.β = −0.11, *p* = 0.010 for R5; S.β = −0.10, *p* = 0.012 for R7). No interaction with age or sex was involved in the association between biological aging clocks and R parameters (Tables S6 and S7).

### 3.4 Moderating Effect of Biological Aging on the Association Between Skin Biomechanics and IC

Since skin elasticity parameters R2, R5, and R7 demonstrated associations with baseline IC and iAgeAA, respectively, we explored whether iAgeAA moderated the relationship between these three R parameters and IC (Table S8). Significant interactions with iAgeAA (i.e., R × iAgeAA and R × age × iAgeAA) were observed for parameters reflecting elastic recovery—R5 (net elasticity) and R7 (biological elasticity). The results suggested that a higher iAgeAA enhanced the effect of R5 and R7 on baseline IC in young and very old individuals. As illustrated in Figure S2, older people with a higher iAgeAA tended to have a lower initial IC when their R5 and R7 parameters were decreased. Conversely, the association between a higher baseline IC and reduced R5 and R7 in younger adults became more pronounced with elevated iAgeAA. Sex‐stratified results are provided in Table S8, where significant associations were primarily observed in women.

## 4 Discussion

This study demonstrated that biomarkers of skin aging—measured through skin biomechanical properties—were associated with functional outcomes across the adult lifespan. Older adults, particularly women, with decreased skin elasticity (R2, R5, R7) and increased viscoelastic part of the deformation (R6) tended to have lower initial IC levels. Higher R6 in older men was associated with faster IC decline over 3 years. On the other hand, the relationship between skin biomechanics and IC was reversed in younger adults. Increased systemic inflammation, indicated by accelerated iAge, was associated with reduced skin elasticity and strengthened the relationship between elastic recovery parameters (R5, R7) and IC.

The association between skin biomechanics and IC in older adults suggested that reduced skin elasticity and viscoelasticity occurred parallel to functional aging. This aligns with the geroscience perspective, which posits that aging‐related pathophysiology in different body organs is connected and represents, at least in part, the consequences and manifestations of the same fundamental mechanisms (Kennedy et al. 2014). In other words, biological aging is a systemic process despite being heterogeneous inter‐individually and intra‐individually (different organs age at different paces). Although this hypothesis warrants further investigation, our findings indicated that skin aging may mirror the holistic aging state of the body (as measured by IC). Evidence from non‐invasive, low‐cost, and less harmful skin markers like elasticity and viscoelasticity is particularly valuable due to its clinical feasibility.

Our analysis of biological aging clocks provides the investigation associating skin markers with the biomarkers derived from the hallmarks of aging, that is, the main biological drivers of the aging process (Kennedy et al. 2014; López‐Otín et al. 2023). Indeed, chronic inflammation and its contributing factors (such as oxidative stress and cellular senescence) act as important mechanisms of intrinsic skin aging (D'Arino et al. 2023; Lemaitre 2024), which could collectively impair skin strength and elasticity by promoting the degradation of collagen and elastin fibers (Pilkington et al. 2021). The accelerated inflammatory age in this study was calculated based on circulating immune proteins, implying that changes in skin biomechanics may result from systemic inflammation. Cutaneous inflammation from a weakened epidermal barrier in aging has also been proposed to contribute back to systemic inflammation (Man and Elias 2019). Although these findings indicate a connection between skin and systemic inflammation, the direction of this relationship remains inconclusive and requires further investigation. The association between the first‐generation epigenetic clocks and skin distensibility (R0) is unknown. Interpreting R0 values can be challenging since both an increase (due to higher immediate extensibility; Chen et al. 2024; Nedelec et al. 2016) and a decrease (indicating improved skin firmness; Chen et al. 2024; Huang et al. 2024) have been linked to a healthy skin condition. In addition, as mentioned above, the non‐standardized nature of R0 makes it less comparable among individuals (Abbas et al. 2022). Additional research is needed to better understand the function of the R0 parameter as an indicator of skin aging.

We observed the reverse association between skin elasticity and IC in younger adults. This paradox may arise because the maximum IC peaked around age 30 (Lu et al. 2023), whereas skin elasticity was suggested to decline from the early 20s (Lephart and Naftolin 2022). Our additional analysis of individual IC trajectories from the baseline to the three‐year visit supports this paradox. As illustrated in Figure S3, many younger participants continued to improve their IC, whereas significant changes in IC were more commonly observed among older people. Furthermore, the current IC metric was primarily evaluated through geriatric assessments (such as MMSE and SPPB), which may have limitations in distinguishing functional differences in younger adults due to ceiling effects in some variables. This is evident from the smaller individual differences in IC trajectories for younger individuals in Figure S3. Finally, this study included a relatively small number of participants aged 20–40 (accounting for 15.4% of the sample). As a result, further research focusing on young individuals is required to clarify the relationship between skin biomechanics and functional capacity in this population, perhaps using maximum tests (e.g., maximum volume of oxygen—${\mathrm{VO}}_{\max}^{2}$) since these tests do not have ceiling effects.

Our study demonstrated how skin mechanical parameters of the Cutometer varied throughout adulthood, using a larger sample than previous research. Consistent with the literature, we found that R7 (biological elasticity) had the strongest correlation with chronological age, supporting its potential to serve as the “age of skin elasticity” (Ahn et al. 2007; Ryu et al. 2008; Chen et al. 2024). Interestingly, this study discovered the longitudinal association with IC only for R6 (viscoelastic ratio). Why R6 outperformed other R parameters in terms of predicting IC evolution remains to be elucidated since higher R6 can result from increased viscous distension, decreased elastic distension, or both (Dobrev 2000). More investigation is also required to understand sex‐specific differences in the longitudinal IC‐R6 association in older adults, namely why R6 predicted IC decline in older men but not women. In addition, studies involving longitudinal and multisite skin elasticity measurements are encouraged to explore how these parameters evolve over time and whether the associations identified in the current studies show regional differences. Furthermore, despite the advantages of being non‐invasive and less harmful, Cutometer data has limitations in providing information at the molecular and cellular levels of the skin. Therefore, additional evidence from skin biopsies is needed to elucidate the connection between systemic and skin‐specific aging. For example, studies incorporating skin biopsy data could use omics techniques to identify aging‐related inflammatory molecules in the skin microenvironment and examine how they interact with the systemic inflammatory clock.

This is the first study exploring the association of skin‐derived biomarkers with global body functions and aging biomarkers, using novel measurements in the field including IC, epigenetic clocks, and iAge. Furthermore, we provided evidence of skin elasticity differences across adulthood in a relatively large, community‐dwelling cohort involving an extended age range and functional status. Nevertheless, some limitations should be raised. First, due to data availability, this study was conducted in a subset of the INSPIRE‐T cohort. Second, only the skin biomechanics of the sun‐unexposed area were assessed in the INSPIRE‐T cohort. Further investigation comparing results from sun‐exposed and sun‐unexposed skin areas is required to provide insight into the role of UV radiation in the relationship between age‐related elasticity decline and IC. Third, the current study lacks data on other aspects of skin biomechanical properties, such as skin induration measured by Durometer and skin echogenicity evaluated via ultrasound. It is plausible that these properties may reveal different relationships with IC and biological aging clocks compared to skin elasticity and viscoelasticity. Fourth, the analysis of skin biomechanics and biological aging clocks was performed using a cross‐sectional design, so we could not establish a causal relationship between them. Yet, the risk of reverse causation is low since skin elasticity is more likely to be a consequence rather than the cause of biological aging. In addition, type I error was expected to increase due to examining several skin parameters and aging biomarkers. Finally, although we controlled for the medical and photoaging factors affecting skin elasticity and viscoelasticity, our findings could be biased by other unmeasured variables, such as skin color (Everett and Sommers 2013) and collagen supplements.

In conclusion, our work demonstrated that skin biomechanical properties may reflect overall functional capacity during aging. Reduced skin elasticity and increased viscoelastic ratio were associated with a lower baseline and an accelerated decline in IC in older adults. Higher levels of systemic inflammation were related to poor skin elasticity, which might serve as the common cause of both organ‐specific (i.e., skin) and whole‐body (i.e., IC) aging. Future research targeting younger individuals and employing suitable functional assessments will provide insight into this topic.

## Author Contributions

W.‐H.L., S.G., P.S.B., and B.V. contributed to the study design. P.S.B. and B.V. supervised the study. S.G. contributed to data collection and verification. S.B.‐T., K.R.H., P.B., and J.L.D. contributed to Cutometer data verification. P.B. and J.‐M.L. managed data of epigenetic clocks. W.‐H.L. analyzed the data. All authors contributed to data interpretation. W.‐H.L. wrote the first version of the manuscript. All authors revised the manuscript with important intellectual content and agreed with the final version to be submitted.

## Conflicts of Interest

P.S.B. declares to have received research grant and consultancy fees from Pfizer. B.V. is the founder president of IHU HealthAge, Toulouse University Hospital, and an investigator in clinical trials sponsored by several industry partners (IHU, CRC, and Inspire gerosicence platforms). S.B.T., K.R.H., P.B., and J.L.D. work for Pierre Fabre Dermo‐Cosmétique & Personal Care. All the other authors declare no conflicts of interest.

## Supporting information


**Figure S1:** Flowchart of sample identification.
**Figure S2:** Interaction plots illustrating the moderating effect of AgeAA on the relationship between IC, R5 (net elasticity) and R7 (biological elasticity) across different ages.
**Figure S3:** Individual IC trajectories over 3 years.
**Table S1:** Descriptive analysis of covariates.
**Table S2:** Conditional effects of R parameters on baseline IC.
**Table S3:** Conditional effects of R parameters on the overtime IC change.
**Table S4:** Linear mixed‐effects model results for individual IC domains.
**Table S5:** Association between R parameters and biological aging clocks.
**Table S6:** Associations of biological age acceleration with R parameters using multiple linear regression included age interaction.
**Table S7:** Associations of biological age acceleration with R parameters using multiple linear regression included sex interaction.
**Table S8:** Summary of coefficients from linear mixed‐effect models for IC, introducing the interaction terms with iAgeAA.

## Data Availability

Request for de‐identified data related to the results reported in this article (i.e., text, tables, figures, and Supporting Information) and a data dictionary will be evaluated by the INSPIRE data‐sharing committee, which can be contacted at the following address: ihuos_inspiredataaccess@chu-toulouse.fr. Data will be made available for investigators upon request for a pre‐identified scientific purpose developed in a research proposal with sound methodology, subject to the approval of the appropriate Committees; a data use agreement must also be signed. Given the ongoing nature of the INSPIRE‐T study, the authors are unable to make the dataset publicly accessible at this time. In addition, according to the INSPIRE policy, all analyses using INSPIRE‐T data (including the present study) must be evaluated and approved by the committee after the submission of a comprehensive analysis proposal. Therefore, for researchers interested in accessing the data used for the current study, the same evaluation procedure must be followed.
