# Supplementary material for: Associations of Skin Biomechanical Properties With Biological Aging Clocks and Longitudinal Changes in Intrinsic Capacity in Adults Aged 20–93: The INSPIRE‐T Project
Source: Aging Cell. 2025 Aug 10;24(10):e70190. doi: 10.1111/acel.70190 (PMC12507415; doi:10.1111/acel.70190)
Supplement: Supplementary file 1 — Figure S1: Flowchart of sample identification. Figure S2: Interaction plots illustrating the moderating effect of AgeAA on the relationship between IC, R5 (net elasticity) and R7 (biological elasticity) across different ages. Figure S3: Individual IC trajectories over 3 years. Table S1: Descriptive analysis of covariates. Table S2: Conditional effects of R parameters on baseline IC. Table S3: Conditional effects of R parameters on the overtime IC change. Table S4: Linear mixed‐effects model results for individual IC domains. Table S5: Association between R parameters and biological aging clocks. Table S6: Associations of biological age acceleration with R parameters using multiple linear regression included age interaction. Table S7: Associations of biological age acceleration with R parameters using multiple linear regression included sex interaction. Table S8: Summary of coefficients from linear mixed‐effect models for IC, introducing the interaction terms with iAgeAA. [file ACEL-24-e70190-s001.docx]

## SUPPORTING INFORMATION

**Methods**

- **Study population: INSPIRE-T cohort**

The INSPIRE-T cohort recruited individuals aged ≥20 (oversampled those aged ≥70 to capture major clinical events), living in the Toulouse area (southwest France), at any level of functional capacity (from robust to frail or disabled) yet without severe illness that may shorten their life expectancy in the short-to-medium term. Participants’ clinical, biological, and imaging data are collected annually (or following the preplanned schedule) according to their willingness for ten years^1^. The INSPIRE-T cohort protocol has been approved by the French Ethical Committee in Rennes (CPP Ouest V). All participants signed informed consent.

- **Epigenetic clocks**

Genomic DNA (gDNA) was extracted from frozen blood samples collected at baseline using the Qiagen DNeasy Blood & Tissue kit. After sample qualification, the gDNA was bisulfite converted, and DNA methylation was profiled according to the manufacturer's instructions using the Illumina EPIC Infinium array (Illumina, Inc). For each CpG locus, methylation levels (β values) ranging from 0 (completely unmethylated) to 1 (completely methylated) were calculated using Partek^®^ Genomics Suite^®^ software. All the β values were then used to calculate the epigenetic ages and acceleration of the epigenetic ages in accordance with the methods recommended in the corresponding literature.

Methylclock R package^2^ was used to calculate Horvath’s pan-tissue clock (353 CpGs^3^), Horvath’s skin & blood clock (391 CpGs^4^), Hannum’s clock (71 CpGs^5^), and PhenoAge (513 CpGs^6^). DNA methylation ages (DNAmAge) and epigenetic age acceleration (AA) parameters were calculated using the default cell count reference panel (“blood gse35069 complete” from Reinius et al.^7^). AA was defined as residuals obtained after regression of chronological age and DNAmAge adjusted for cell number. Blood cell types were estimated from DNA methylation data using the algorithm with the “blood gse35069 complete” reference panel in Reinius et al^7^. This panel was constructed from the methylation profiles of seven isolated cell types: CD4+ T cells, CD8+ T cells, CD56+ NK cells, CD19+ B cells, CD14+ monocytes, neutrophils, and eosinophils. No cell types were measured directly in the laboratory; all proportions were deduced by calculation.

We calculated DNAmGrimAge and the residual of DNAmGrimAge adjusted on age (i.e., AgeAccelGrim) as described previously^8^.

DunedinPACE is an epigenetic clock developed to quantify the rate of biological aging from blood test. It is based on the analysis of 19 biomarkers of organ systems monitored longitudinally as part of the Dunedin study^9^. DunedinPACE was calculated in accordance with the authors’ recommendations, as briefly outlined below. IDAT files were processed in R (v4.4.3). The methylation data were functionally normalized (FunNorm) using the minfi software package and used for downstream calculations. DunedinPACE scores were calculated using the dedicated R package DunedinPACE (v0.99.0). The proportionOfProbes Required parameter was set at 0.7, in line with the authors’ recommendations for EPIC chips.

- **Covariates: Factors related to sun exposure**

*Previous residence in high daily solar exposure regions (a binary variable):* Participants were categorized as having lived in high daily solar exposure regions (defined as Zone 4 or higher in the below figure) if they reported living in these regions for several months or longer.

**
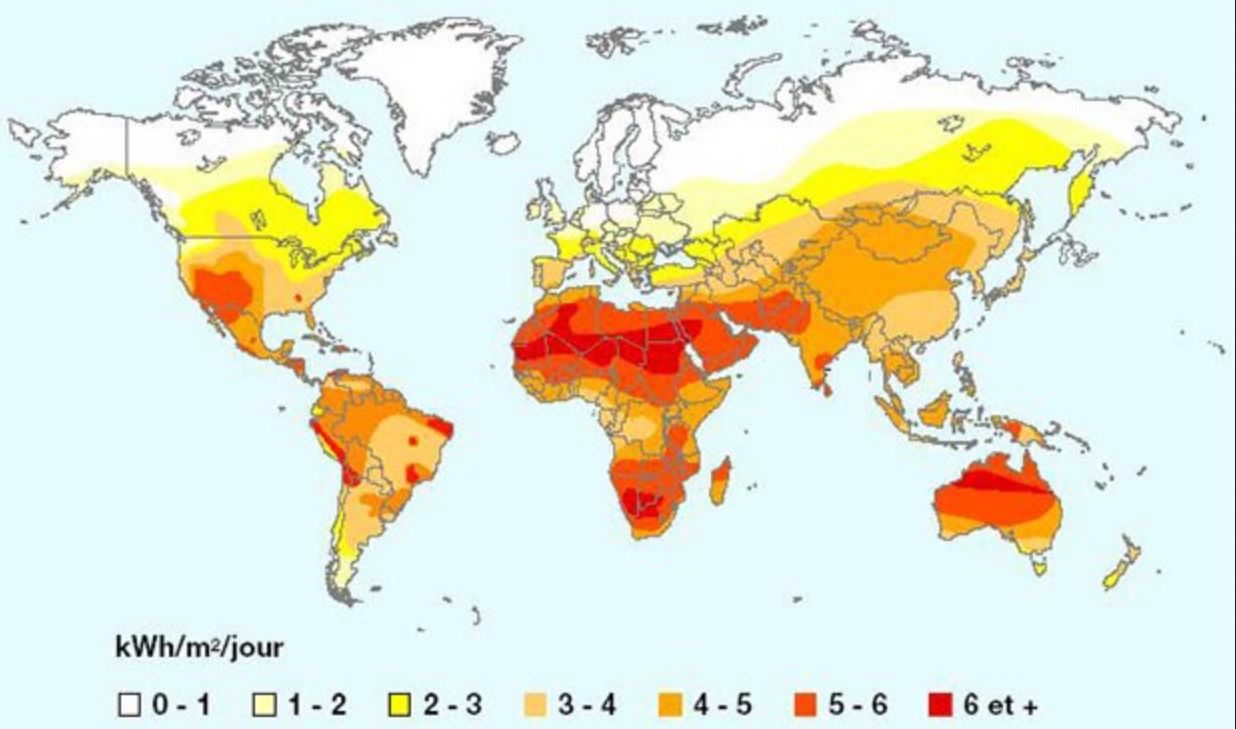
**

*Use of ultraviolet (UV) protection products:* Participants were asked if they protected their lower limbs during sun exposure with sun protection products (never use, sun protection factor [SPF]<50, or SPF 50).

*Frequency of sun exposure on the lower limbs:* Participants were asked about the frequency of sun exposure on their lower limbs in four scenarios: during vacation before and after age 15, and outside vacation before and after age 15. Vacation exposure frequency was scored as 0 (never), 1 (one week/year), 2 (two weeks/year), 3 (three weeks/year), 4 (four to five weeks/year), or 5 (six weeks or more/year), while outside vacation exposure was scored as 0 (never), 1 (some days per year), 2 (some days per month), 3 (some days per week), or 4 (daily). Based on four scenarios, a total score ranging from 0 to 18 was used for analysis, with higher scores indicating a higher frequency of sun exposure.

**References**

1. Guyonnet S, Rolland Y, Takeda C, et al. The INSPIRE Bio-resource Research Platform for Healthy Aging and Geroscience: Focus on the Human Translational Research Cohort (The INSPIRE-T Cohort). J Frailty Aging. 2020;10(2):1-11. doi:10.14283/jfa.2020.38
2. Pelegí-Sisó D, de Prado P, Ronkainen J, Bustamante M, González JR. methylclock: a Bioconductor package to estimate DNA methylation age. Bioinformatics. 2021;37(12):1759-1760. doi:10.1093/bioinformatics/btaa825
3. Horvath S. DNA methylation age of human tissues and cell types. Genome Biol. 2013;14(10):1-20. doi:10.1186/gb-2013-14-10-r115
4. Horvath S, Oshima J, Martin GM, et al. Epigenetic clock for skin and blood cells applied to Hutchinson Gilford Progeria Syndrome and ex vivo studies. Aging (Albany NY). 2018;10(7):1758-1775. doi:10.18632/aging.101508
5. Hannum G, Guinney J, Zhao L, et al. Genome-wide Methylation Profiles Reveal Quantitative Views of Human Aging Rates. Mol Cell. 2013;49(2):359-367. doi:10.1016/j.molcel.2012.10.016
6. Levine ME, Lu AT, Quach A, et al. An epigenetic biomarker of aging for lifespan and healthspan. Aging (Albany NY). 2018;10(4):573-591. doi:10.18632/aging.101414
7. Reinius LE, Acevedo N, Joerink M, et al. Differential DNA methylation in purified human blood cells: Implications for cell lineage and studies on disease susceptibility. PLoS One. 2012;7(7):e41361. doi:10.1371/journal.pone.0041361
8. Lu AT, Quach A, Wilson JG, et al. DNA methylation GrimAge strongly predicts lifespan and healthspan. Aging (Albany NY). 2019;11(2):303-327. doi:10.18632/aging.101684
9. Belsky DW, Caspi A, Corcoran DL, et al. DunedinPACE, A DNA methylation biomarker of the Pace of Aging. Elife. 2022;11:e73420. doi:10.7554/eLife.73420

**Figure S1. Flowchart of sample identification**

**
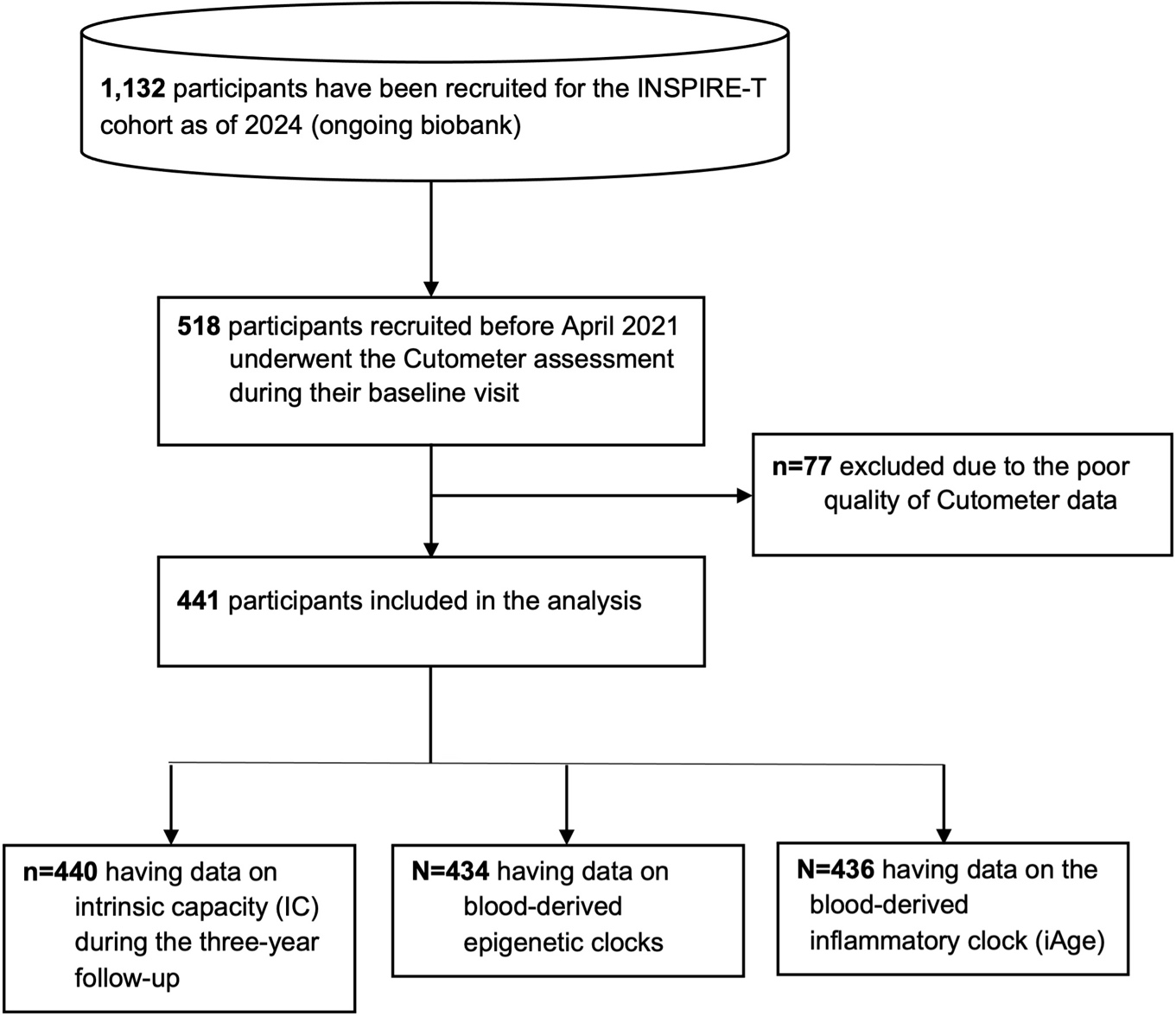
**

**Figure S2. Interaction plots illustrating the moderating effect of AgeAA on the relationship between IC, R5 (net elasticity), and R7 (biological elasticity) across different ages.**


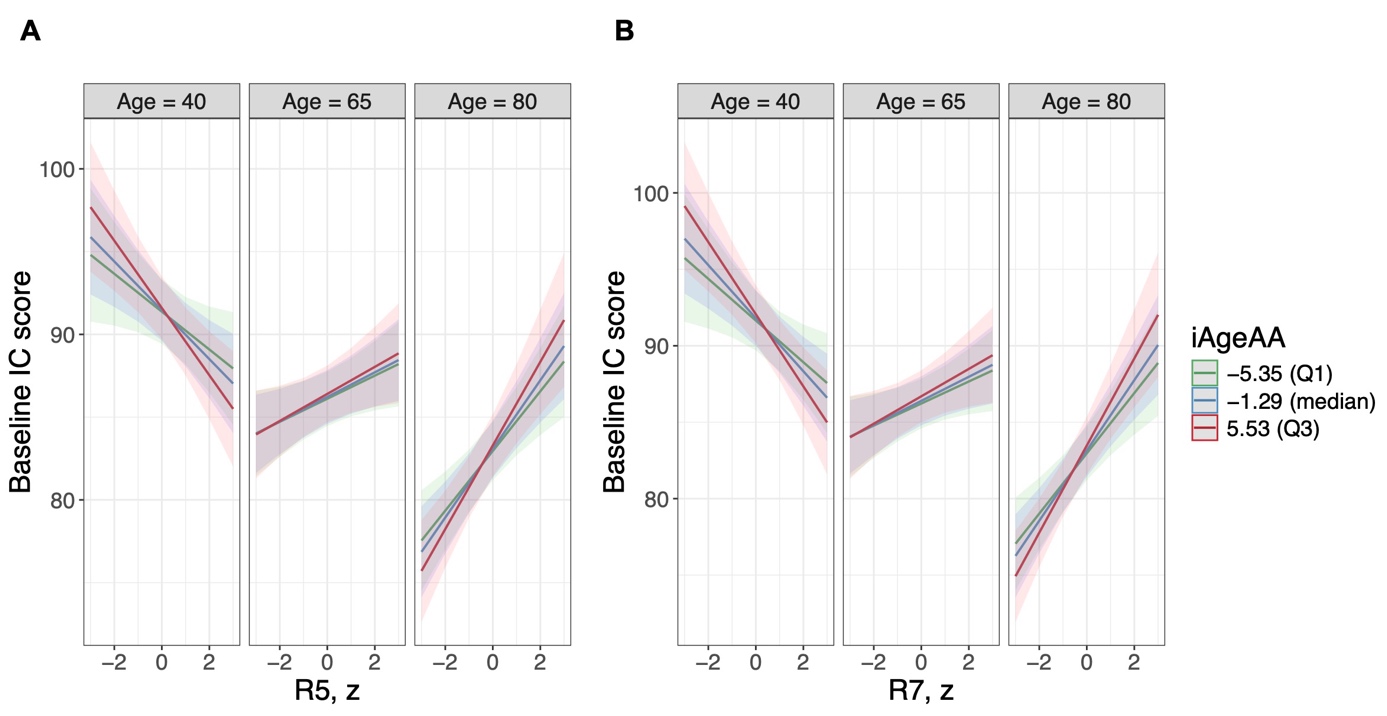


The interaction plots illustrate how the association between baseline IC and the skin elasticity parameters R5 **(A)** and R7 **(B)** varied with the levels of age acceleration in iAge (iAgeAA) in the whole population, using 40, 65, and 80 years as examples. The slopes of the R parameters were estimated at time = 0, with ages of 40, 65, and 80 and iAgeAA values of Q1 (-5.35 years), median (-1.29 years), and Q3 (5.53 years), respectively, based on the linear mixed-effect models presented in Table S8. These ages and iAgeAA values were selected to visualize the age-moderating effects better. A higher iAgeAA tended to enhance the impact of R5 and R7 on baseline IC in younger and older individuals. For instance, the red line, representing individuals with an iAgeAA equal to the Q3 value, exhibited a steeper slope in the plots than those with lower iAgeAA (the blue and green lines) for individuals aged 40 and 80.

**Figure S3. Individual IC trajectories over three years**

Spaghetti plots show the actual individual IC trajectories over the three-year follow-up, colored according to their R6 values (viscoelastic ratio; in standardized z-score). For better clarity, the figures display only subjects’ baseline and third-year data, plotted according to their ages at the respective time points. The plots aim to visualize the significant association between higher R6 and faster IC decline in older individuals, as steeper, declining IC trajectories tend to have higher R6 (colored dark purple).

**Table S1. Descriptive analysis of covariates**

|  | **Whole population**  **(n=441)** | **Women**  **(n=264)** | **Men**  **(n=177)** |
| --- | --- | --- | --- |
| Hormone replacement therapy (HRT) user | 11 (2.5%) | 11 (4.2%) | - |
| Previous residence in high solar exposure regions (n=438) | 99 (22.6%) | 48 (18.2%) | 51 (29.3%) |
| Use of UV protection products (n=437) |  |  |  |
| Never | 228 (52.2%) | 102 (39.1%) | 126 (71.6%) |
| SPF <50 | 72 (16.5%) | 56 (21.5%) | 16 (9.1%) |
| SPF 50 | 137 (31.4%) | 103 (39.5%) | 34 (19.3%) |
| Frequency of sun exposure, total score 0-18 (n=438) | 10.9 (3.9) | 10.0 (4.1) | 12.2 (3.2) |
| Before age 15, during vacation (n=439) |  |  |  |
| Never | 37 (8.4%) | 32 (12.2%) | 5 (2.8%) |
| 1 week per year | 17 (3.9%) | 13 (4.9%) | 4 (2.3%) |
| 2 weeks per year | 21 (4.8%) | 15 (5.7%) | 6 (3.4%) |
| 3 weeks per year | 42 (9.6%) | 30 (11.4%) | 12 (6.8%) |
| 4-5 weeks per year | 88 (20.1%) | 64 (24.3%) | 24 (13.6%) |
| 6+ weeks per year | 234 (53.3%) | 109 (41.4%) | 125 (71.0%) |
| After age 15, during vacation (n=439) |  |  |  |
| Never | 11 (2.5%) | 10 (3.8%) | 1 (0.6%) |
| 1 week per year | 30 (6.8%) | 18 (6.9%) | 12 (6.8%) |
| 2 weeks per year | 63 (14.4%) | 44 (16.8%) | 19 (10.7%) |
| 3 weeks per year | 71 (16.2%) | 45 (17.2%) | 26 (14.7%) |
| 4-5 weeks per year | 121 (27.6%) | 75 (28.6%) | 46 (26.0%) |
| 6+ weeks per year | 143 (32.6%) | 70 (26.7%) | 73 (41.2%) |
| Before age 15, outside vacation (n=440) |  |  |  |
| Never | 79 (18.0%) | 62 (23.6%) | 17 (9.6%) |
| Some days per year | 99 (22.5%) | 70 (26.6%) | 29 (16.4%) |
| Some days per month | 145 (33.0%) | 81 (30.8%) | 64 (36.2%) |
| Some days per week | 67 (15.2%) | 32 (12.2%) | 35 (19.8%) |
| Daily | 50 (11.4%) | 18 (6.8%) | 32 (18.1%) |
| After age 15, outside vacation (n=440) |  |  |  |
| Never | 59 (13.4%) | 41 (15.6%) | 18 (10.2%) |
| Some days per year | 133 (30.2%) | 81 (30.8%) | 52 (29.4%) |
| Some days per month | 174 (39.6%) | 103 (39.2%) | 71 (40.1%) |
| Some days per week | 57 (13.0%) | 31 (11.8%) | 26 (14.7%) |
| Daily | 17 (3.9%) | 7 (2.7%) | 10 (5.7%) |

Values are displayed as mean (SD) or number (%). SPF, sun protection factor; UV, ultraviolet.

**Table S2. Conditional effects of R parameters on baseline IC**

| **R parameter** | **Sample** | **Age interval where the effect of R on baseline IC is significant** | **Conditional effects of R on baseline IC at the age interval of significance** | | | |
| --- | --- | --- | --- | --- | --- | --- |
|  |  |  | **β** | **Lower limit of the 95% CI** | **Upper limit of the 95% CI** | **p** |
| R0 (skin distensibility) | Whole population | ns | | | | |
|  | Women | ns | | | | |
|  | Men | ns | | | | |
| R2 (gross elasticity) | Whole population | [20, 48]  [64, 93] | [-3.11, -0.76]  [0.58, 3.02] | [-4.56, -1.50]  [0.03, 2.00] | [-1.67, -0.02]  [1.14, 4.04] | [<0.001, 0.044] [0.038, <0.001] |
|  | Women | [20, 45]  [61, 93] | [-3.90, -1.07]  [0.74, 4.37] | [-5.72, -2.08]  [0.03, 3.04] | [-2.08, -0.06]  [1.46, 5.71] | [<0.001, 0.038] [0.042, <0.001] |
|  | Men | ns | | | | |
| R5 (net elasticity) | Whole population | [20, 50]  [64, 93] | [-3.55, -0.71]  [0.61, 3.34] | [-4.92, -1.39]  [0.03, 2.26] | [-2.17, -0.04]  [1.18, 4.43] | [<0.001, 0.039] [0.039, <0.001] |
|  | Women | [20, 47]  [62, 93] | [-4.14, -0.98]  [0.78, 4.41] | [-5.85, -1.88]  [0.04, 2.99] | [-2.43, -0.07]  [1.52, 5.83] | [<0.001, 0.034]  [0.038, <0.001] |
|  | Men | [20, 40] | [-2.65, -1.48] | [-4.96, -2.96] | [-0.34, -0.01] | [0.024, 0.049] |
| R6 (viscoelastic ratio) | Whole population | [20, 45]  [71, 93] | [2.47, 0.94]  [-0.66, -2.00] | [0.84, 0.00]  [-1.26, -3.02] | [4.10, 1.87]  [-0.05, -0.99] | [0.003, 0.049]  [0.035, <0.001] |
|  | Women | [20, 46]  [70, 93] | [3.47, 1.22]  [-0.86, -2.85] | [1.40, 0.05]  [-1.69, -4.22] | [5.55, 2.40]  [-0.02, -1.48] | [0.001, 0.041]  [0.045, <0.001] |
|  | Men | ns | | | | |
| R7 (biological elasticity) | Whole population | [20, 51]  [64, 93] | [-4.07, -0.75]  [0.65, 3.76] | [-5.43, -1.44]  [0.04, 2.68] | [-2.71, -0.06]  [1.26, 4.83] | [<0.001, 0.034] [0.038, <0.001] |
|  | Women | [20, 49]  [62, 93] | [-4.62, -0.89]  [0.78, 4.77] | [-6.29, -1.77]  [0.02, 3.40] | [-2.95, -0.00]  [1.55, 6.14] | [<0.001, 0.049]  [0.454, <0.001] |
|  | Men | [20, 44]  [89, 93] | [-3.01, -1.41]  [1.59, 1.86] | [-5.33, -2.79]  [0.03, 0.14] | [-0.68, -0.03]  [3.16, 3.58] | [0.011, 0.452]  [0.046, 0.034] |

Age interval of significance was identified using the Johnson-Neyman technique. β represents the change in baseline IC score for a one-SD increase in the R parameter, as estimated using the adjusted models in Table 2 (for the whole population) and Table 3 (for women and men) with the time variable set to 0 and age fixed at the boundary of the significance interval. ns, non-significant association between the R parameter and IC at baseline.

**Table S3. Conditional effects of R parameters on the overtime IC change**

| **R parameter** | **Sample** | **Age interval where the effect of R on time is significant** | **Conditional effects of R on time at the age interval of significance** | | | |
| --- | --- | --- | --- | --- | --- | --- |
|  |  |  | **β** | **Lower limit of the 95% CI** | **Upper limit of the 95% CI** | **p** |
| R0 (skin distensibility) | Whole population | [20, 43] | [-0.56, -0.28] | [-1.05, -0.56] | [-0.08, -0.00] | [0.022, 0.048] |
|  | Women | ns | | | | |
|  | Men | ns | | | | |
| R2 (gross elasticity) | Whole population | ns | | | | |
|  | Women | ns | | | | |
|  | Men | ns | | | | |
| R5 (net elasticity) | Whole population | ns | | | | |
|  | Women | ns | | | | |
|  | Men | ns | | | | |
| R6 (viscoelastic ratio) | Whole population | [20, 47]  [75, 93] | [0.88, 0.32]  [-0.25, -0.62] | [0.28, 0.00]  [-0.50, -1.01] | [1.48, 0.64]  [-0.01, -0.23] | [0.004, 0.048]  [0.043, 0.002] |
|  | Women | [20, 66] | [0.91, 0.31] | [0.18, 0.01] | [1.64, 0.61] | [0.015, 0.042] |
|  | Men | [62, 93] | [-0.37, -1.32] | [-0.72, -1.91] | [-0.03, -0.73] | [0.033, <0.001] |
| R7 (biological elasticity) | Whole population | ns | | | | |
|  | Women | ns | | | | |
|  | Men | ns | | | | |

Age interval of significance was identified using the Johnson-Neyman technique. β represents the change in the slope of time on IC for one SD increase in the R parameter, as estimated using the adjusted models in Table 2 (for the whole population) and Table 3 (for women and men), with age fixed at the boundary of the significance interval. ns, non-significant association between the R parameter and overtime IC change.

**Table S4. Linear mixed-effects model results for individual IC domains**

|  | **R0 (skin distensibility)** | | | **R2 (gross elasticity)** | | | **R5 (net elasticity)** | | | **R6 (viscoelastic ratio)** | | | **R7 (biological elasticity)** | | |
| --- | --- | --- | --- | --- | --- | --- | --- | --- | --- | --- | --- | --- | --- | --- | --- |
|  | **β** | **S.E.** | **p** | **β** | **S.E.** | **p** | **β** | **S.E.** | **p** | **β** | **S.E.** | **p** | **β** | **S.E.** | **p** |
| **Outcome: Cognition** |  |  |  |  |  |  |  |  |  |  |  |  |  |  |  |
| **R** | -0.07 | 0.20 | 0.739 | -1.66 | 0.66 | 0.012 | -1.71 | 0.63 | 0.007 | -0.17 | 0.21 | 0.422 | -1.89 | 0.63 | 0.003 |
| **age** | -0.08 | 0.01 | <0.001 | -0.07 | 0.01 | <0.001 | -0.07 | 0.01 | <0.001 | -0.08 | 0.01 | <0.001 | -0.07 | 0.01 | <0.001 |
| **R × age** | - | - | - | 0.03 | 0.01 | 0.007 | 0.03 | 0.01 | 0.004 | - | - | - | 0.03 | 0.01 | 0.001 |
| **time** | 0.35 | 0.08 | <0.001 | 0.34 | 0.08 | <0.001 | 0.34 | 0.08 | <0.001 | 0.44 | 0.08 | <0.001 | 0.34 | 0.08 | <0.001 |
| **time × R** | -0.04 | 0.08 | 0.571 | 0.17 | 0.08 | 0.032 | 0.11 | 0.08 | 0.157 | 0.68 | 0.24 | 0.006 | 0.12 | 0.08 | 0.134 |
| **time × age** | - | - | - | - | - | - | - | - | - | -0.01 | 0.00 | 0.002 | - | - | - |
| **time × R × age** | - | - | - | - | - | - | - | - | - | - | - | - | - | - | - |
| **Outcome: Locomotion** |  |  |  |  |  |  |  |  |  |  |  |  |  |  |  |
| **R** | -0.28 | 0.41 | 0.499 | -2.94 | 1.62 | 0.069 | -3.59 | 1.56 | 0.022 | 5.42 | 1.65 | 0.001 | -4.35 | 1.54 | 0.005 |
| **age** | -0.15 | 0.02 | <0.001 | -0.13 | 0.03 | <0.001 | -0.14 | 0.03 | <0.001 | -0.18 | 0.03 | <0.001 | -0.14 | 0.03 | <0.001 |
| **R × age** | - | - | - | 0.06 | 0.02 | 0.015 | 0.06 | 0.02 | 0.007 | -0.09 | 0.02 | <0.001 | 0.08 | 0.02 | 0.001 |
| **time** | 2.10 | 0.63 | 0.001 | 1.56 | 0.62 | 0.012 | 1.68 | 0.66 | 0.011 | 3.09 | 0.78 | <0.001 | 1.72 | 0.71 | 0.015 |
| **time × R** | 0.11 | 0.17 | 0.508 | -1.11 | 0.67 | 0.098 | -1.19 | 0.65 | 0.067 | 1.76 | 0.70 | 0.012 | -1.41 | 0.64 | 0.027 |
| **time × age** | -0.04 | 0.01 | <0.001 | -0.03 | 0.01 | 0.001 | -0.03 | 0.01 | 0.001 | -0.06 | 0.01 | <0.001 | -0.03 | 0.01 | 0.003 |
| **time × R × age** | - | - | - | 0.03 | 0.01 | 0.010 | 0.03 | 0.01 | 0.008 | -0.03 | 0.01 | 0.006 | 0.03 | 0.01 | 0.002 |
| **Outcome: Psychology** |  |  |  |  |  |  |  |  |  |  |  |  |  |  |  |
| **R** | 0.32 | 0.55 | 0.567 | -6.73 | 2.00 | 0.001 | -7.09 | 1.93 | <0.001 | -0.53 | 0.59 | 0.366 | -6.70 | 1.91 | <0.001 |
| **age** | -0.01 | 0.03 | 0.796 | 0.01 | 0.03 | 0.801 | -0.01 | 0.03 | 0.757 | 0.00 | 0.03 | 0.906 | -0.02 | 0.03 | 0.496 |
| **R × age** | - | - | - | 0.10 | 0.03 | 0.001 | 0.10 | 0.03 | 0.001 | - | - | - | 0.10 | 0.03 | 0.001 |
| **time** | -0.29 | 0.21 | 0.164 | -0.29 | 0.21 | 0.159 | -0.3 | 0.21 | 0.155 | -0.28 | 0.21 | 0.180 | -0.30 | 0.21 | 0.155 |
| **time × R** | -0.48 | 0.21 | 0.019 | 0.29 | 0.21 | 0.160 | 0.39 | 0.21 | 0.061 | 0.20 | 0.21 | 0.355 | 0.37 | 0.21 | 0.077 |
| **time × age** | - | - | - | - | - | - | - | - | - | - | - | - | - | - | - |
| **time × R × age** | - | - | - | - | - | - | - | - | - | - | - | - | - | - | - |
| **Outcome: Vitality** |  |  |  |  |  |  |  |  |  |  |  |  |  |  |  |
| **R** | -5.16 | 1.89 | 0.007 | -6.61 | 2.22 | 0.003 | -6.53 | 2.14 | 0.002 | 7.32 | 2.14 | 0.001 | -7.78 | 2.11 | <0.001 |
| **age** | -0.47 | 0.03 | <0.001 | -0.40 | 0.03 | <0.001 | -0.42 | 0.04 | <0.001 | -0.47 | 0.04 | <0.001 | -0.42 | 0.04 | <0.001 |
| **R × age** | 0.08 | 0.03 | 0.005 | 0.12 | 0.03 | <0.001 | 0.12 | 0.03 | <0.001 | -0.13 | 0.03 | <0.001 | 0.14 | 0.03 | <0.001 |
| **time** | 3.27 | 0.55 | <0.001 | 3.74 | 0.55 | <0.001 | 3.77 | 0.57 | <0.001 | 3.26 | 0.59 | <0.001 | 3.88 | 0.62 | <0.001 |
| **time × R** | 0.04 | 0.15 | 0.809 | 1.09 | 0.59 | 0.063 | 0.94 | 0.57 | 0.097 | -0.04 | 0.16 | 0.794 | 0.67 | 0.56 | 0.234 |
| **time × age** | -0.06 | 0.01 | <0.001 | -0.07 | 0.01 | <0.001 | -0.07 | 0.01 | <0.001 | -0.06 | 0.01 | <0.001 | -0.07 | 0.01 | <0.001 |
| **time × R × age** | - | - | - | -0.02 | 0.01 | 0.009 | -0.02 | 0.01 | 0.013 | - | - | - | -0.02 | 0.01 | 0.036 |
| **Outcome: Sensory** |  |  |  |  |  |  |  |  |  |  |  |  |  |  |  |
| **R** | -0.65 | 0.80 | 0.417 | -7.89 | 2.63 | 0.003 | -9.87 | 2.52 | <0.001 | 8.39 | 3.24 | 0.010 | -11.77 | 2.47 | <0.001 |
| **age** | -0.54 | 0.04 | <0.001 | -0.48 | 0.04 | <0.001 | -0.47 | 0.04 | <0.001 | -0.57 | 0.06 | <0.001 | -0.48 | 0.04 | <0.001 |
| **R × age** | - | - | - | 0.14 | 0.04 | <0.001 | 0.19 | 0.04 | <0.001 | -0.12 | 0.05 | 0.012 | 0.22 | 0.04 | <0.001 |
| **time** | -0.04 | 0.32 | 0.901 | -0.08 | 0.32 | 0.798 | -0.08 | 0.32 | 0.806 | 2.65 | 1.48 | 0.073 | -0.09 | 0.32 | 0.779 |
| **time × R** | 0.14 | 0.32 | 0.657 | 0.66 | 0.32 | 0.041 | 0.45 | 0.32 | 0.164 | 2.46 | 1.33 | 0.065 | 0.60 | 0.32 | 0.065 |
| **time × age** | - | - | - | - | - | - | - | - | - | -0.04 | 0.02 | 0.084 | - | - | - |
| **time × R × age** | - | - | - | - | - | - | - | - | - | -0.04 | 0.02 | 0.021 | - | - | - |

The values of R parameters were first standardized to have a mean of 0 and a SD of 1 before performing the linear mixed-effect models, allowing for comparisons across models. A year was used as the unit of time. The models were additionally adjusted for sex, use of hormone replacement therapy (HRT), previous residence in high-solar-exposure regions, use of ultraviolet (UV) protection products, and sun exposure frequency. Interaction terms that were not retained in the models due to lack of significance are indicated by “-“.

**Table S5. Association between R parameters and biological aging clocks**

|  | **Unadjusted models** | | | | **Adjusted models ^a^** | | | |
| --- | --- | --- | --- | --- | --- | --- | --- | --- |
|  | **β** | **S.E.** | **S.β** | **p** | **β** | **S.E.** | **S.β** | **p** |
| **Outcome: R0 (skin distensibility)** |  |  |  |  |  |  |  |  |
| HorvathAA pan-tissue | 9E-04 | 1E-03 | 0.04 | 0.410 | 3E-03 | 1E-03 | 0.12 | 0.014 |
| HorvathAA skin-blood | -9E-05 | 2E-03 | 0.00 | 0.955 | 4E-03 | 2E-03 | 0.12 | 0.023 |
| HannumAA | 6E-04 | 1E-03 | 0.02 | 0.677 | 3E-03 | 1E-03 | 0.09 | 0.053 |
| PhenoAA | -2E-04 | 1E-03 | -0.01 | 0.867 | 8E-04 | 1E-03 | 0.04 | 0.440 |
| GrimAA | -4E-03 | 1E-03 | -0.14 | 0.003 | -3E-03 | 1E-03 | -0.10 | 0.056 |
| DunedinPACE | -7E-02 | 4E-02 | -0.08 | 0.081 | -8E-02 | 4E-02 | -0.09 | 0.068 |
| iAgeAA | -5E-05 | 6E-04 | 0.00 | 0.939 | -6E-04 | 6E-04 | -0.05 | 0.317 |
| **Outcome: R2 (gross elasticity)** |  |  |  |  |  |  |  |  |
| HorvathAA pan-tissue | 1E-03 | 2E-03 | 0.04 | 0.377 | -1E-03 | 1E-03 | -0.03 | 0.496 |
| HorvathAA skin-blood | 6E-03 | 2E-03 | 0.13 | 0.009 | 1E-03 | 2E-03 | 0.03 | 0.580 |
| HannumAA | -1E-03 | 2E-03 | -0.02 | 0.639 | -1E-03 | 2E-03 | -0.03 | 0.494 |
| PhenoAA | 1E-03 | 1E-03 | 0.04 | 0.402 | 6E-04 | 1E-03 | 0.02 | 0.676 |
| GrimAA | 8E-04 | 2E-03 | 0.02 | 0.683 | 2E-03 | 2E-03 | 0.04 | 0.377 |
| DunedinPACE | 1E-02 | 6E-02 | 0.01 | 0.814 | -6E-03 | 5E-02 | 0.00 | 0.910 |
| iAgeAA | -2E-03 | 8E-04 | -0.13 | 0.005 | -2E-03 | 8E-04 | -0.10 | 0.018 |
| **Outcome: R5 (net elasticity)** |  |  |  |  |  |  |  |  |
| HorvathAA pan-tissue | 1E-03 | 2E-03 | 0.02 | 0.613 | -2E-03 | 2E-03 | -0.04 | 0.422 |
| HorvathAA skin-blood | 6E-03 | 3E-03 | 0.10 | 0.045 | 1E-03 | 3E-03 | 0.02 | 0.619 |
| HannumAA | -1E-03 | 3E-03 | -0.02 | 0.650 | -9E-04 | 3E-03 | -0.02 | 0.725 |
| PhenoAA | 2E-03 | 2E-03 | 0.06 | 0.250 | 2E-03 | 2E-03 | 0.05 | 0.265 |
| GrimAA | 2E-04 | 3E-03 | 0.00 | 0.932 | 2E-03 | 3E-03 | 0.03 | 0.519 |
| DunedinPACE | 8E-02 | 8E-02 | 0.04 | 0.351 | 6E-02 | 7E-02 | 0.04 | 0.405 |
| iAgeAA | -3E-03 | 1E-03 | -0.12 | 0.016 | -3E-03 | 1E-03 | -0.11 | 0.010 |
| **Outcome: R6 (viscoelastic ratio)** |  |  |  |  |  |  |  |  |
| HorvathAA pan-tissue | 8E-04 | 1E-03 | 0.03 | 0.525 | 8E-04 | 1E-03 | 0.03 | 0.499 |
| HorvathAA skin-blood | -6E-04 | 2E-03 | -0.02 | 0.745 | -2E-03 | 2E-03 | -0.05 | 0.226 |
| HannumAA | 9E-04 | 2E-03 | 0.02 | 0.608 | -4E-04 | 2E-03 | -0.01 | 0.818 |
| PhenoAA | 8E-04 | 1E-03 | 0.03 | 0.498 | 3E-04 | 1E-03 | 0.01 | 0.787 |
| GrimAA | 2E-03 | 2E-03 | 0.06 | 0.217 | 2E-03 | 2E-03 | 0.05 | 0.250 |
| DunedinPACE | 8E-02 | 5E-02 | 0.08 | 0.092 | 8E-02 | 4E-02 | 0.08 | 0.062 |
| iAgeAA | -6E-04 | 7E-04 | -0.05 | 0.333 | -3E-04 | 6E-04 | -0.02 | 0.634 |
| **Outcome: R7 (biological elasticity)** |  |  |  |  |  |  |  |  |
| HorvathAA pan-tissue | 4E-04 | 2E-03 | 0.01 | 0.785 | -1E-03 | 1E-03 | -0.04 | 0.370 |
| HorvathAA skin-blood | 4E-03 | 2E-03 | 0.09 | 0.062 | 2E-03 | 2E-03 | 0.04 | 0.344 |
| HannumAA | -1E-03 | 2E-03 | -0.03 | 0.542 | -5E-04 | 2E-03 | -0.01 | 0.804 |
| PhenoAA | 1E-03 | 1E-03 | 0.05 | 0.337 | 1E-03 | 1E-03 | 0.05 | 0.229 |
| GrimAA | -5E-04 | 2E-03 | -0.01 | 0.808 | 8E-04 | 2E-03 | 0.02 | 0.665 |
| DunedinPACE | 3E-02 | 6E-02 | 0.03 | 0.587 | 2E-02 | 5E-02 | 0.02 | 0.670 |
| iAgeAA | -2E-03 | 8E-04 | -0.10 | 0.044 | -2E-03 | 7E-04 | -0.10 | 0.012 |

^a^ Models were adjusted for age, age^2^ (except for R6), sex, under hormone replacement therapy (HRT), previous residence in high-solar-exposure regions, use of ultraviolet (UV) protection products, and sun exposure frequency. S.β: standardized regression coefficient, which is the regression coefficient β multiplied by the SD of predictor (i.e., biomarkers) and divided by the SD of outcome (i.e., R parameters).

**Table S6. Associations of biological age acceleration with R parameters using multiple linear regression included age interaction**

| **Predictors** | **Outcomes** | | | | | | | | | | | | | | | | | | | |
| --- | --- | --- | --- | --- | --- | --- | --- | --- | --- | --- | --- | --- | --- | --- | --- | --- | --- | --- | --- | --- |
|  | **R0 (skin distensibility)** | | | | **R2 (gross elasticity)** | | | | **R5 (net elasticity)** | | | | **R6 (viscoelastic ratio)** | | | | **R7 (biological elasticity)** | | | |
|  | **β** | **S.E.** | **S.β** | **p** | **β** | **S.E.** | **S.β** | **p** | **β** | **S.E.** | **S.β** | **p** | **β** | **S.E.** | **S.β** | **p** | **β** | **S.E.** | **S.β** | **p** |
| ***HorvathAA pan-tissue*** |  |  |  |  |  |  |  |  |  |  |  |  |  |  |  |  |  |  |  |  |
| **HorvathAA** | 1E-02 | 1E-02 | 0.46 | 0.306 | -6E-03 | 1E-02 | -0.20 | 0.641 | -6E-03 | 2E-02 | -0.14 | 0.740 | -5E-04 | 4E-03 | -0.02 | 0.898 | -3E-03 | 1E-02 | -0.08 | 0.825 |
| **age** | -7E-03 | 2E-03 | -1.21 | <0.001 | 1E-02 | 2E-03 | 1.72 | <0.001 | 1E-02 | 3E-03 | 1.29 | <0.001 | 3E-03 | 3E-04 | 0.48 | <0.001 | 8E-03 | 2E-03 | 0.97 | <0.001 |
| **age^2^** | 5E-05 | 2E-05 | 0.99 | 0.002 | -1E-04 | 2E-05 | -2.04 | <0.001 | -2E-04 | 3E-05 | -1.72 | <0.001 | - | - | - | - | -1E-04 | 2E-05 | -1.51 | <0.001 |
| **HorvathAA × age** | -3E-04 | 4E-04 | -0.79 | 0.461 | 2E-05 | 5E-04 | 0.05 | 0.963 | 2E-05 | 7E-04 | 0.03 | 0.977 | 2E-05 | 6E-05 | 0.05 | 0.748 | -4E-05 | 4E-04 | -0.09 | 0.927 |
| **HorvathAA × age^2^** | 2E-06 | 3E-06 | 0.47 | 0.488 | 8E-07 | 4E-06 | 0.13 | 0.839 | 7E-07 | 6E-06 | 0.08 | 0.900 | - | - | - | - | 9E-07 | 4E-06 | 0.14 | 0.808 |
| ***HorvathAA skin-blood*** |  |  |  |  |  |  |  |  |  |  |  |  |  |  |  |  |  |  |  |  |
| **HorvathAA** | -7E-04 | 2E-02 | -0.02 | 0.965 | -2E-02 | 2E-02 | -0.38 | 0.408 | -3E-03 | 3E-02 | -0.05 | 0.916 | -4E-03 | 6E-03 | -0.10 | 0.543 | 2E-03 | 2E-02 | 0.04 | 0.915 |
| **age** | -7E-03 | 2E-03 | -1.19 | 0.001 | 1E-02 | 3E-03 | 1.73 | <0.001 | 1E-02 | 4E-03 | 1.18 | <0.001 | 3E-03 | 3E-04 | 0.49 | <0.001 | 7E-03 | 3E-03 | 0.79 | 0.012 |
| **age^2^** | 5E-05 | 2E-05 | 0.98 | 0.006 | -1E-04 | 2E-05 | -2.04 | <0.001 | -2E-04 | 3E-05 | -1.62 | <0.001 | - | - | - | - | -1E-04 | 2E-05 | -1.35 | <0.001 |
| **HorvathAA × age** | 1E-04 | 6E-04 | 0.29 | 0.801 | 5E-04 | 8E-04 | 0.71 | 0.510 | 5E-05 | 1E-03 | 0.05 | 0.960 | 3E-05 | 9E-05 | 0.05 | 0.763 | -3E-05 | 7E-04 | -0.04 | 0.971 |
| **HorvathAA × age^2^** | -1E-06 | 5E-06 | -0.16 | 0.828 | -3E-06 | 6E-06 | -0.32 | 0.635 | 3E-07 | 9E-06 | 0.02 | 0.977 | - | - | - | - | 3E-07 | 6E-06 | 0.04 | 0.954 |
| ***HannumAA*** |  |  |  |  |  |  |  |  |  |  |  |  |  |  |  |  |  |  |  |  |
| **HannumAA** | 2E-03 | 2E-02 | 0.07 | 0.891 | -2E-02 | 2E-02 | -0.37 | 0.465 | -1E-02 | 3E-02 | -0.22 | 0.660 | -1E-03 | 7E-03 | -0.04 | 0.848 | -2E-02 | 2E-02 | -0.34 | 0.463 |
| **age** | -6E-03 | 2E-03 | -0.98 | 0.001 | 1E-02 | 2E-03 | 1.66 | <0.001 | 1E-02 | 3E-03 | 1.22 | <0.001 | 3E-03 | 3E-04 | 0.48 | <0.001 | 8E-03 | 2E-03 | 0.93 | <0.001 |
| **age^2^** | 4E-05 | 2E-05 | 0.78 | 0.008 | -1E-04 | 2E-05 | -2.00 | <0.001 | -2E-04 | 3E-05 | -1.67 | <0.001 | - | - | - | - | -1E-04 | 2E-05 | -1.49 | <0.001 |
| **HannumAA × age** | -2E-04 | 6E-04 | -0.48 | 0.699 | 5E-04 | 8E-04 | 0.80 | 0.496 | 5E-04 | 1E-03 | 0.56 | 0.620 | 1E-05 | 1E-04 | 0.03 | 0.887 | 6E-04 | 7E-04 | 0.85 | 0.423 |
| **HannumAA × age^2^** | 3E-06 | 5E-06 | 0.52 | 0.483 | -4E-06 | 6E-06 | -0.47 | 0.507 | -5E-06 | 9E-06 | -0.37 | 0.585 | - | - | - | - | -5E-06 | 6E-06 | -0.54 | 0.400 |
| ***PhenoAA*** |  |  |  |  |  |  |  |  |  |  |  |  |  |  |  |  |  |  |  |  |
| **PhenoAA** | -2E-03 | 1E-02 | -0.10 | 0.832 | -4E-03 | 1E-02 | -0.14 | 0.756 | 5E-04 | 2E-02 | 0.01 | 0.978 | 2E-03 | 4E-03 | 0.10 | 0.542 | 1E-03 | 1E-02 | 0.04 | 0.917 |
| **age** | -5E-03 | 2E-03 | -0.92 | 0.003 | 1E-02 | 2E-03 | 1.57 | <0.001 | 1E-02 | 3E-03 | 1.11 | <0.001 | 3E-03 | 3E-04 | 0.47 | <0.001 | 7E-03 | 2E-03 | 0.81 | 0.002 |
| **age^2^** | 4E-05 | 2E-05 | 0.73 | 0.017 | -1E-04 | 2E-05 | -1.93 | <0.001 | -2E-04 | 3E-05 | -1.59 | <0.001 | - | - | - | - | -1E-04 | 2E-05 | -1.38 | <0.001 |
| **PhenoAA × age** | -3E-05 | 4E-04 | -0.08 | 0.942 | 4E-04 | 5E-04 | 0.90 | 0.404 | 4E-04 | 7E-04 | 0.63 | 0.546 | -3E-05 | 6E-05 | -0.09 | 0.576 | 2E-04 | 5E-04 | 0.52 | 0.597 |
| **PhenoAA × age^2^** | 1E-06 | 3E-06 | 0.24 | 0.742 | -5E-06 | 4E-06 | -0.78 | 0.251 | -5E-06 | 6E-06 | -0.62 | 0.341 | - | - | - | - | -3E-06 | 4E-06 | -0.54 | 0.382 |
| ***GrimAA*** |  |  |  |  |  |  |  |  |  |  |  |  |  |  |  |  |  |  |  |  |
| **GrimAA** | -2E-02 | 2E-02 | -0.80 | 0.207 | 3E-02 | 2E-02 | 0.81 | 0.175 | 6E-02 | 3E-02 | 1.00 | 0.083 | 6E-03 | 6E-03 | 0.18 | 0.319 | 3E-02 | 2E-02 | 0.78 | 0.153 |
| **age** | -5E-03 | 2E-03 | -0.91 | 0.002 | 1E-02 | 2E-03 | 1.59 | <0.001 | 1E-02 | 3E-03 | 1.17 | <0.001 | 3E-03 | 3E-04 | 0.48 | <0.001 | 7E-03 | 2E-03 | 0.87 | <0.001 |
| **age^2^** | 4E-05 | 1E-05 | 0.69 | 0.016 | -1E-04 | 2E-05 | -1.94 | <0.001 | -2E-04 | 3E-05 | -1.62 | <0.001 | - | - | - | - | -1E-04 | 2E-05 | -1.43 | <0.001 |
| **GrimAA × age** | 6E-04 | 6E-04 | 1.25 | 0.370 | -1E-03 | 8E-04 | -1.75 | 0.185 | -2E-03 | 1E-03 | -2.10 | 0.100 | -7E-05 | 9E-05 | -0.13 | 0.468 | -1E-03 | 8E-04 | -1.65 | 0.169 |
| **GrimAA × age^2^** | -4E-06 | 5E-06 | -0.56 | 0.491 | 9E-06 | 7E-06 | 1.02 | 0.184 | 1E-05 | 9E-06 | 1.17 | 0.117 | - | - | - | - | 9E-06 | 7E-06 | 0.92 | 0.186 |
| ***DunedinPACE*** |  |  |  |  |  |  |  |  |  |  |  |  |  |  |  |  |  |  |  |  |
| **DunedinPACE** | -3E-01 | 4E-01 | -0.29 | 0.499 | 8E-02 | 5E-01 | 0.06 | 0.879 | 2E-01 | 7E-01 | 0.14 | 0.722 | 1E-01 | 1E-01 | 0.10 | 0.489 | 1E-01 | 5E-01 | 0.10 | 0.795 |
| **age** | -1E-02 | 1E-02 | -1.72 | 0.464 | 1E-02 | 2E-02 | 1.54 | 0.488 | 2E-02 | 2E-02 | 1.89 | 0.378 | 3E-03 | 2E-03 | 0.53 | 0.120 | 1E-02 | 2E-02 | 1.56 | 0.440 |
| **age^2^** | 6E-05 | 1E-04 | 1.23 | 0.607 | -1E-04 | 2E-04 | -1.56 | 0.490 | -2E-04 | 2E-04 | -2.44 | 0.265 | - | - | - | - | -2E-04 | 2E-04 | -2.29 | 0.264 |
| **DunedinPACE × age** | 5E-03 | 1E-02 | 0.84 | 0.741 | 5E-04 | 2E-02 | 0.06 | 0.980 | -9E-03 | 3E-02 | -0.76 | 0.742 | -4E-04 | 2E-03 | -0.06 | 0.880 | -6E-03 | 2E-02 | -0.73 | 0.738 |
| **DunedinPACE × age^2^** | -3E-05 | 1E-04 | -0.51 | 0.836 | -3E-05 | 2E-04 | -0.39 | 0.866 | 9E-05 | 2E-04 | 0.82 | 0.714 | - | - | - | - | 7E-05 | 2E-04 | 0.87 | 0.678 |
| ***iAgeAA*** |  |  |  |  |  |  |  |  |  |  |  |  |  |  |  |  |  |  |  |  |
| **iAgeAA** | -5E-04 | 6E-03 | -0.04 | 0.935 | -1E-03 | 7E-03 | -0.06 | 0.878 | 3E-03 | 1E-02 | 0.12 | 0.773 | -2E-03 | 2E-03 | -0.12 | 0.475 | 6E-04 | 7E-03 | 0.03 | 0.935 |
| **age** | -6E-03 | 2E-03 | -0.95 | 0.001 | 1E-02 | 2E-03 | 1.55 | <0.001 | 1E-02 | 3E-03 | 1.15 | <0.001 | 3E-03 | 3E-04 | 0.47 | <0.001 | 7E-03 | 2E-03 | 0.84 | <0.001 |
| **age^2^** | 4E-05 | 2E-05 | 0.73 | 0.013 | -1E-04 | 2E-05 | -1.89 | <0.001 | -2E-04 | 3E-05 | -1.59 | <0.001 | - | - | - | - | -1E-04 | 2E-05 | -1.40 | <0.001 |
| **iAgeAA × age** | 3E-05 | 2E-04 | 0.15 | 0.887 | 1E-06 | 3E-04 | 0.00 | 0.997 | -2E-04 | 4E-04 | -0.43 | 0.651 | 2E-05 | 4E-05 | 0.10 | 0.541 | -6E-05 | 2E-04 | -0.21 | 0.818 |
| **iAgeAA × age^2^** | -5E-07 | 2E-06 | -0.17 | 0.794 | -2E-07 | 2E-06 | -0.05 | 0.938 | 1E-06 | 3E-06 | 0.21 | 0.722 | - | - | - | - | 3E-07 | 2E-06 | 0.08 | 0.894 |

Models were additionally adjusted for sex, under hormone replacement therapy (HRT), previous residence in high-solar-exposure regions, use of ultraviolet (UV) protection products, and sun exposure frequency. S.β: standardized regression coefficient, multiplied the regression coefficient β by the SD of predictor and dividing by the SD of outcome.

**Table S7. Associations of biological age acceleration with R parameters using multiple linear regression included sex interaction**

| **Predictors** | **Outcomes** | | | | | | | | | | | | | | | | | | | |
| --- | --- | --- | --- | --- | --- | --- | --- | --- | --- | --- | --- | --- | --- | --- | --- | --- | --- | --- | --- | --- |
|  | **R0 (skin distensibility)** | | | | **R2 (gross elasticity)** | | | | **R5 (net elasticity)** | | | | **R6 (viscoelastic ratio)** | | | | **R7 (biological elasticity)** | | | |
|  | **β** | **S.E.** | **S.β** | **p** | **β** | **S.E.** | **S.β** | **p** | **β** | **S.E.** | **S.β** | **p** | **β** | **S.E.** | **S.β** | **p** | **β** | **S.E.** | **S.β** | **p** |
| ***HorvathAA pan-tissue*** |  |  |  |  |  |  |  |  |  |  |  |  |  |  |  |  |  |  |  |  |
| **HorvathAA** | 1E-03 | 1E-03 | 0.06 | 0.305 | -2E-03 | 2E-03 | -0.06 | 0.348 | -2E-03 | 3E-03 | -0.05 | 0.412 | 1E-03 | 1E-03 | 0.04 | 0.507 | -2E-03 | 2E-03 | -0.06 | 0.258 |
| **sex** | -4E-02 | 1E-02 | -0.19 | <0.001 | 2E-03 | 1E-02 | 0.01 | 0.884 | -7E-03 | 2E-02 | -0.02 | 0.721 | -6E-03 | 1E-02 | -0.03 | 0.605 | -6E-03 | 1E-02 | -0.02 | 0.657 |
| **HorvathAA × sex** | 3E-03 | 2E-03 | 0.09 | 0.158 | 2E-03 | 3E-03 | 0.04 | 0.514 | 1E-03 | 4E-03 | 0.02 | 0.755 | -5E-04 | 2E-03 | -0.01 | 0.825 | 2E-03 | 3E-03 | 0.04 | 0.489 |
| ***HorvathAA skin-blood*** |  |  |  |  |  |  |  |  |  |  |  |  |  |  |  |  |  |  |  |  |
| **HorvathAA** | 2E-03 | 2E-03 | 0.05 | 0.443 | 3E-03 | 3E-03 | 0.06 | 0.311 | 3E-03 | 4E-03 | 0.05 | 0.374 | -9E-04 | 2E-03 | -0.02 | 0.663 | 3E-03 | 3E-03 | 0.06 | 0.266 |
| **sex** | -4E-02 | 1E-02 | -0.18 | <0.001 | 2E-04 | 1E-02 | 0.00 | 0.988 | -1E-02 | 2E-02 | -0.02 | 0.608 | -2E-03 | 1E-02 | -0.01 | 0.838 | -9E-03 | 1E-02 | -0.03 | 0.498 |
| **HorvathAA × sex** | 6E-03 | 3E-03 | 0.11 | 0.074 | -4E-03 | 4E-03 | -0.05 | 0.340 | -5E-03 | 6E-03 | -0.05 | 0.412 | -3E-03 | 3E-03 | -0.05 | 0.420 | -2E-03 | 4E-03 | -0.03 | 0.552 |
| ***HannumAA*** |  |  |  |  |  |  |  |  |  |  |  |  |  |  |  |  |  |  |  |  |
| **HannumAA** | 2E-03 | 2E-03 | 0.06 | 0.336 | 7E-04 | 3E-03 | 0.02 | 0.770 | 3E-03 | 3E-03 | 0.05 | 0.374 | 6E-04 | 2E-03 | 0.02 | 0.761 | 2E-03 | 2E-03 | 0.04 | 0.435 |
| **sex** | -4E-02 | 1E-02 | -0.18 | <0.001 | 2E-03 | 1E-02 | 0.01 | 0.907 | -9E-03 | 2E-02 | -0.02 | 0.644 | -4E-03 | 1E-02 | -0.02 | 0.733 | -8E-03 | 1E-02 | -0.03 | 0.555 |
| **HannumAA × sex** | 2E-03 | 3E-03 | 0.05 | 0.418 | -5E-03 | 4E-03 | -0.07 | 0.205 | -1E-02 | 5E-03 | -0.10 | 0.072 | -2E-03 | 3E-03 | -0.04 | 0.459 | -6E-03 | 4E-03 | -0.08 | 0.132 |
| ***PhenoAA*** |  |  |  |  |  |  |  |  |  |  |  |  |  |  |  |  |  |  |  |  |
| **PhenoAA** | 9E-04 | 1E-03 | 0.04 | 0.507 | 6E-06 | 2E-03 | 0.00 | 0.997 | 2E-03 | 2E-03 | 0.05 | 0.417 | -2E-04 | 1E-03 | -0.01 | 0.888 | 1E-03 | 2E-03 | 0.05 | 0.377 |
| **sex** | -4E-02 | 1E-02 | -0.17 | 0.001 | 2E-04 | 1E-02 | 0.00 | 0.989 | -1E-02 | 2E-02 | -0.03 | 0.550 | -4E-03 | 1E-02 | -0.02 | 0.714 | -1E-02 | 1E-02 | -0.03 | 0.473 |
| **PhenoAA × sex** | -3E-04 | 2E-03 | -0.01 | 0.894 | 1E-03 | 3E-03 | 0.03 | 0.643 | 1E-04 | 4E-03 | 0.00 | 0.975 | 1E-03 | 2E-03 | 0.03 | 0.608 | 6E-05 | 2E-03 | 0.00 | 0.982 |
| ***GrimAA*** |  |  |  |  |  |  |  |  |  |  |  |  |  |  |  |  |  |  |  |  |
| **GrimAA** | -8E-04 | 2E-03 | -0.03 | 0.699 | -1E-03 | 3E-03 | -0.03 | 0.635 | -5E-04 | 4E-03 | -0.01 | 0.878 | 7E-04 | 2E-03 | 0.02 | 0.735 | -4E-04 | 2E-03 | -0.01 | 0.860 |
| **sex** | -2E-02 | 1E-02 | -0.11 | 0.050 | -1E-02 | 2E-02 | -0.04 | 0.505 | -2E-02 | 2E-02 | -0.05 | 0.367 | -1E-02 | 1E-02 | -0.05 | 0.362 | -1E-02 | 1E-02 | -0.04 | 0.374 |
| **GrimAA × sex** | -4E-03 | 3E-03 | -0.11 | 0.135 | 6E-03 | 4E-03 | 0.11 | 0.100 | 5E-03 | 5E-03 | 0.06 | 0.359 | 2E-03 | 3E-03 | 0.05 | 0.459 | 3E-03 | 4E-03 | 0.04 | 0.468 |
| ***DunedinPACE*** |  |  |  |  |  |  |  |  |  |  |  |  |  |  |  |  |  |  |  |  |
| **DunedinPACE** | -1E-01 | 5E-02 | -0.11 | 0.060 | 3E-02 | 7E-02 | 0.03 | 0.634 | 1E-01 | 9E-02 | 0.06 | 0.274 | 1E-01 | 6E-02 | 0.11 | 0.042 | 5E-02 | 7E-02 | 0.04 | 0.467 |
| **sex** | -9E-02 | 8E-02 | -0.44 | 0.245 | 9E-02 | 1E-01 | 0.32 | 0.371 | 9E-02 | 1E-01 | 0.22 | 0.527 | 7E-02 | 8E-02 | 0.31 | 0.381 | 5E-02 | 1E-01 | 0.18 | 0.589 |
| **DunedinPACE × sex** | 6E-02 | 9E-02 | 0.28 | 0.463 | -1E-01 | 1E-01 | -0.33 | 0.367 | -1E-01 | 2E-01 | -0.25 | 0.478 | -8E-02 | 9E-02 | -0.33 | 0.349 | -7E-02 | 1E-01 | -0.21 | 0.528 |
| ***iAgeAA*** |  |  |  |  |  |  |  |  |  |  |  |  |  |  |  |  |  |  |  |  |
| **iAgeAA** | -7E-04 | 8E-04 | -0.05 | 0.382 | -1E-03 | 1E-03 | -0.07 | 0.211 | -2E-03 | 1E-03 | -0.08 | 0.150 | -4E-05 | 8E-04 | 0.00 | 0.964 | -1E-03 | 9E-04 | -0.08 | 0.137 |
| **sex** | -4E-02 | 1E-02 | -0.17 | 0.001 | -2E-03 | 1E-02 | -0.01 | 0.859 | -2E-02 | 2E-02 | -0.04 | 0.392 | -7E-03 | 1E-02 | -0.03 | 0.527 | -1E-02 | 1E-02 | -0.04 | 0.355 |
| **iAgeAA × sex** | 2E-04 | 1E-03 | 0.01 | 0.875 | -1E-03 | 2E-03 | -0.05 | 0.351 | -2E-03 | 2E-03 | -0.05 | 0.384 | -6E-04 | 1E-03 | -0.03 | 0.614 | -1E-03 | 1E-03 | -0.04 | 0.459 |

Models were additionally adjusted for age, age^2^ (except for R6), under hormone replacement therapy (HRT), previous residence in high-solar-exposure regions, use of ultraviolet (UV) protection products, and sun exposure frequency. S.β: standardized regression coefficient, multiplied the regression coefficient β by the SD of predictor and dividing by the SD of outcome.

**Table S8. Summary of coefficients from linear mixed-effect models for IC, introducing the interaction terms with iAgeAA**

|  | **Whole population ^a^** | | | **Women ^b^** | | | **Men ^c^** | | |
| --- | --- | --- | --- | --- | --- | --- | --- | --- | --- |
|  | **β** | **S.E.** | **p** | **β** | **S.E.** | **p** | **β** | **S.E.** | **p** |
| ***R2 (gross elasticity)*** |  |  |  |  |  |  |  |  |  |
| **R** | -4.61 | 1.04 | <0.001 | -6.00 | 1.32 | <0.001 | -2.82 | 1.76 | 0.110 |
| **iAgeAA** | -0.02 | 0.13 | 0.892 | 0.10 | 0.16 | 0.556 | -0.08 | 0.21 | 0.696 |
| **R × iAgeAA** | -0.14 | 0.13 | 0.302 | -0.12 | 0.18 | 0.503 | -0.17 | 0.21 | 0.438 |
| **age** | -0.20 | 0.02 | <0.001 | -0.19 | 0.02 | <0.001 | -0.20 | 0.03 | <0.001 |
| **age × iAgeAA** | 0.00 | 0.00 | 0.850 | 0.00 | 0.00 | 0.369 | 0.00 | 0.00 | 0.447 |
| **R × age** | 0.08 | 0.02 | <0.001 | 0.11 | 0.02 | <0.001 | 0.04 | 0.03 | 0.090 |
| **R × age × iAgeAA** | 0.00 | 0.00 | 0.259 | 0.00 | 0.00 | 0.567 | 0.00 | 0.00 | 0.388 |
| **time** | 1.46 | 0.38 | <0.001 | 1.31 | 0.46 | 0.004 | 1.77 | 0.68 | 0.010 |
| **time × iAgeAA** | -0.02 | 0.05 | 0.720 | -0.05 | 0.06 | 0.390 | 0.03 | 0.08 | 0.721 |
| **time × R** | 0.16 | 0.11 | 0.142 | 0.13 | 0.14 | 0.346 | 0.21 | 0.18 | 0.247 |
| **time × R × iAgeAA** | 0.01 | 0.01 | 0.669 | 0.00 | 0.02 | 0.913 | 0.01 | 0.02 | 0.465 |
| **time × age** | -0.03 | 0.01 | <0.001 | -0.02 | 0.01 | 0.001 | -0.03 | 0.01 | 0.004 |
| **time × age × iAgeAA** | 0.00 | 0.00 | 0.839 | 0.00 | 0.00 | 0.399 | 0.00 | 0.00 | 0.607 |
| ***R5 (net elasticity)*** |  |  |  |  |  |  |  |  |  |
| **R** | -5.32 | 1.00 | <0.001 | -6.61 | 1.26 | <0.001 | -4.27 | 1.80 | 0.018 |
| **iAgeAA** | 0.01 | 0.13 | 0.938 | 0.19 | 0.17 | 0.259 | -0.11 | 0.21 | 0.612 |
| **R × iAgeAA** | -0.23 | 0.12 | 0.064 | -0.34 | 0.17 | 0.044 | -0.11 | 0.21 | 0.590 |
| **age** | -0.21 | 0.02 | <0.001 | -0.21 | 0.02 | <0.001 | -0.20 | 0.03 | <0.001 |
| **age × iAgeAA** | 0.00 | 0.00 | 0.901 | 0.00 | 0.00 | 0.216 | 0.00 | 0.00 | 0.341 |
| **R × age** | 0.09 | 0.02 | <0.001 | 0.12 | 0.02 | <0.001 | 0.07 | 0.03 | 0.009 |
| **R × age × iAgeAA** | 0.00 | 0.00 | 0.048 | 0.00 | 0.00 | 0.057 | 0.00 | 0.00 | 0.468 |
| **time** | 1.44 | 0.40 | <0.001 | 1.31 | 0.48 | 0.006 | 1.71 | 0.72 | 0.018 |
| **time × iAgeAA** | -0.02 | 0.05 | 0.610 | -0.05 | 0.06 | 0.381 | 0.02 | 0.08 | 0.799 |
| **time × R** | 0.12 | 0.11 | 0.289 | 0.08 | 0.14 | 0.570 | 0.18 | 0.19 | 0.352 |
| **time × R × iAgeAA** | 0.01 | 0.01 | 0.494 | 0.00 | 0.02 | 0.986 | 0.02 | 0.02 | 0.306 |
| **time × age** | -0.03 | 0.01 | <0.001 | -0.02 | 0.01 | 0.002 | -0.03 | 0.01 | 0.010 |
| **time × age × iAgeAA** | 0.00 | 0.00 | 0.719 | 0.00 | 0.00 | 0.397 | 0.00 | 0.00 | 0.687 |
| ***R7 (biological elasticity)*** |  |  |  |  |  |  |  |  |  |
| **R** | -6.11 | 0.97 | <0.001 | -7.39 | 1.22 | <0.001 | -4.65 | 1.76 | 0.009 |
| **iAgeAA** | 0.03 | 0.14 | 0.831 | 0.23 | 0.18 | 0.199 | -0.11 | 0.21 | 0.611 |
| **R × iAgeAA** | -0.26 | 0.12 | 0.034 | -0.37 | 0.17 | 0.024 | -0.17 | 0.21 | 0.424 |
| **age** | -0.22 | 0.02 | <0.001 | -0.22 | 0.02 | <0.001 | -0.20 | 0.03 | <0.001 |
| **age × iAgeAA** | 0.00 | 0.00 | 0.917 | 0.00 | 0.00 | 0.192 | 0.00 | 0.00 | 0.301 |
| **R × age** | 0.11 | 0.01 | <0.001 | 0.13 | 0.02 | <0.001 | 0.08 | 0.03 | 0.003 |
| **R × age × iAgeAA** | 0.00 | 0.00 | 0.020 | 0.01 | 0.00 | 0.031 | 0.00 | 0.00 | 0.275 |
| **time** | 1.36 | 0.43 | 0.002 | 1.38 | 0.51 | 0.007 | 1.32 | 0.76 | 0.084 |
| **time × iAgeAA** | -0.02 | 0.05 | 0.630 | -0.06 | 0.07 | 0.379 | 0.03 | 0.08 | 0.744 |
| **time × R** | 0.15 | 0.12 | 0.234 | 0.03 | 0.15 | 0.866 | 0.36 | 0.21 | 0.084 |
| **time × R × iAgeAA** | 0.01 | 0.01 | 0.603 | 0.00 | 0.02 | 0.877 | 0.01 | 0.02 | 0.564 |
| **time × age** | -0.02 | 0.01 | <0.001 | -0.03 | 0.01 | 0.002 | -0.02 | 0.01 | 0.056 |
| **time × age × iAgeAA** | 0.00 | 0.00 | 0.733 | 0.00 | 0.00 | 0.405 | 0.00 | 0.00 | 0.656 |

Analyses were performed on participants having available data on IC and biological aging clocks. Values of R parameters were first standardized as mean=0 and SD=1 before performing linear mixed-effect models. ^a^ Models were additionally adjusted for sex, under hormone replacement therapy (HRT), previous residence in high-solar-exposure regions, use of ultraviolet (UV) protection products, and sun exposure frequency. ^b^ Models were additionally adjusted for under HRT, previous residence in high-solar-exposure regions, use of UV protection products, and sun exposure frequency. ^c^ Models were additionally adjusted for previous residence in high-solar-exposure regions, use of UV protection products, and sun exposure frequency.
